# Survalytics: An Open-Source Cloud-Integrated Experience Sampling, Survey, and Analytics and Metadata Collection Module for Android Operating System Apps

**DOI:** 10.2196/mhealth.5397

**Published:** 2016-06-03

**Authors:** Vikas O'Reilly-Shah, Sean Mackey

**Affiliations:** ^1^ Assistant Professor of Anesthesiology Emory University and Children's Healthcare of Atlanta Atlanta, GA United States; ^2^ Redlich Professor of Anesthesiology, Perioperative and Pain Medicine, Neurosciences and Neurology Stanford University Stanford, CA United States

**Keywords:** experiential sampling, ecological momentary assessment, quantified self, analytics, Android, Amazon Web Services, DynamoDB, NoSQL, surveys, microsurveys, mobile surveys

## Abstract

**Background:**

We describe here Survalytics, a software module designed to address two broad areas of need. The first area is in the domain of surveys and app analytics: developers of mobile apps in both academic and commercial environments require information about their users, as well as how the apps are being used, to understand who their users are and how to optimally approach app development. The second area of need is in the field of ecological momentary assessment, also referred to as experience sampling: researchers in a wide variety of fields, spanning from the social sciences to psychology to clinical medicine, would like to be able to capture daily or even more frequent data from research subjects while in their natural environment.

**Objective:**

Survalytics is an open-source solution for the collection of survey responses as well as arbitrary analytic metadata from users of Android operating system apps.

**Methods:**

Surveys may be administered in any combination of one-time questions and ongoing questions. The module may be deployed as a stand-alone app for experience sampling purposes or as an add-on to existing apps. The module takes advantage of free-tier NoSQL cloud database management offered by the Amazon Web Services DynamoDB platform to package a secure, flexible, extensible data collection module. DynamoDB is capable of Health Insurance Portability and Accountability Act compliant storage of personal health information.

**Results:**

The provided example app may be used without modification for a basic experience sampling project, and we provide example questions for daily collection of blood glucose data from study subjects.

**Conclusions:**

The module will help researchers in a wide variety of fields rapidly develop tailor-made Android apps for a variety of data collection purposes.

## Introduction

The explosion of cheap mobile computing technology has led to rapid global adoption of smartphone and tablet technology, with annual smartphone sales accounting for the majority of mobile phones worldwide by 2013 [1]. The Android operating system (OS) is loaded onto more than 75% of these smartphones [1,2]. Accompanying the mobile computing revolution has been the explosion of apps available for mobile OSs, with 1.6 million apps available in Google Play for the Android OS as of July 2015 [3].

The profusion of mobile phones has put vast processing power into the pockets of a large swath of the population. Thus, successful apps with a large number of users may experience commercial success, but also provide an opportunity for studying population-level behavioral dynamics with sample sizes and data precision heretofore impossible to collect.

The corresponding author has written an app, “Anesthesiologist,” for the Android OS platform that has experienced a moderate degree of success, with 100,000 users from around the world as of late 2015 [4]. In planning and deploying a research study related to this app, we developed a reusable module for the Android OS that fills 2 broad areas of need.

First, this research study highlighted the need for a tool that allows dual collection of survey data as well as analytics. This allows us to take the analytics data, and behavioral aspects of app use related to these analytics, and parse it on the basis of usage by specific subpopulations. Other developers may be interested in combining analytics with survey data to inform their development cycle. Finally, commercial app developers are particularly interested in characterizing the demographics and usage patterns of their user base for targeted advertising purposes. There are several private commercial solutions that can provide some of the functionality of this module in terms of survey administration [5-8]. However, as mature as some of these products are, none are open source and all are fee-for-service. Moreover, none appear to have the flexibility and extensibility to allow the capture of arbitrary app analytics, requiring 2 vendor solutions to combine analytics with survey administration.

The ability to combine survey and analytic data on a per-user basis is the basis on which investigators can understand how particular subpopulations are using their app. Using “Anesthesiologist” as an example, we know that the app is used by health care practitioners in a wide variety of roles, but we need to use surveys to differentiate the attending anesthesiologist from the junior level trainee. We are also interested in understanding the frequency of app usage, which we can capture using the analytics piece. However, to test the hypothesis that junior level trainees use the app more frequently than attendings, we need to combine this data. The ability to combine analytics with survey data allows investigators to perform a much more nuanced analysis of incoming data.

The second need served by this project is in the area of ecological momentary assessment [9-11], also referred to as experiential sampling. Experience sampling procedures have been used for many years to facilitate the collection of data from subjects during the normal course of their day, as opposed to collection of this data during visits to an office or a lab. This reduces biases in the data related to the need for subjects to remember or aggregate their experiences over a certain interval [12]. These procedures also allow subjects to answer questions within their natural environment as well, perhaps reducing error or bias related to being in the laboratory environment [12]. Quantified-self movement enthusiasts likewise wish to take advantage of modern technology in the pursuit of “self-knowledge through numbers” [13-15].

Several modern experience sampling tools have been developed and released [16-18]. In this arena, mature products have significant costs associated with their use. These include SurveySignal [19], Diario [20], LifeData [21], mEMA [22], iForm [23], movisensXS [24], and iSurvey [25]. Even the relatively mature open-source solutions do not implement enterprise database solutions to provide extensibility, scalability, and security. Paco [26], Aware [27,28], and funf [29] require setup of stand-alone servers, with their attendant information technology (IT) and security concerns as well as the associated cost of hardware and maintenance. Emotion sense [27] and Purple Robot [30] do not offer a storage solution, only export of JSON.

Survalytics represents a substantial improvement over these packages by solving the backend database problem by integrating with Amazon Web Services (AWS) DynamoDB. Amazon Web Services DynamoDB is a hosted NoSQL database service with a flexible data model. For the requirements of Survalytics, minimal database setup is required (described in Multimedia Appendix 1). At the time of writing, AWS offers *indefinite* free-tier service on DynamoDB [31], with 25 GB of free storage and enough free throughput to handle 200 million requests per month. This represents far more capacity than most projects will ever require.

Effective question delivery and response storage is impossible without a solution to this the backend database problem. Compared with stand-alone server solutions, AWS offers intrinsically better security at zero cost, with physical security at the AWS server farms, enterprise-level security architecture and monitoring, active feature development and resource management by AWS, and fine-grained access management options. Amazon Web Services also offers the advantage of virtually guaranteed uptime and extremely low access latency. Survalytics was partially built based on our experience with the development of an unreleased Android port of the Experience Sampling Program, a now antiquated ecological momentary assessment solution built for Palm OS that suffered from many of these problems [12,32].

In short, Survalytics represents a significant leap forward for open-source experience sampling solutions and fills an unmet need in the research community by combining analytics with one-time and ongoing survey question administration on a popular, cheap, widely available OS platform with integration of a cloud-based database service. We expect this combination will fill an important need in the research community by serving broad swaths of investigators interested in app analytics and administration of one-time surveys to their user base as well as those interested in ecological momentary assessment.

## Methods

### Software Functionalities

The 3 major functionalities of the module are as follows:

1. Stand-alone app for experience sampling (ongoing questions) and one-time survey administration
2. Add-on module for the administration of surveys to users of existing Android apps
3. Add-on module for the collection of arbitrary data from app interactions or background processes

### Software Architecture

**Figure 1:**
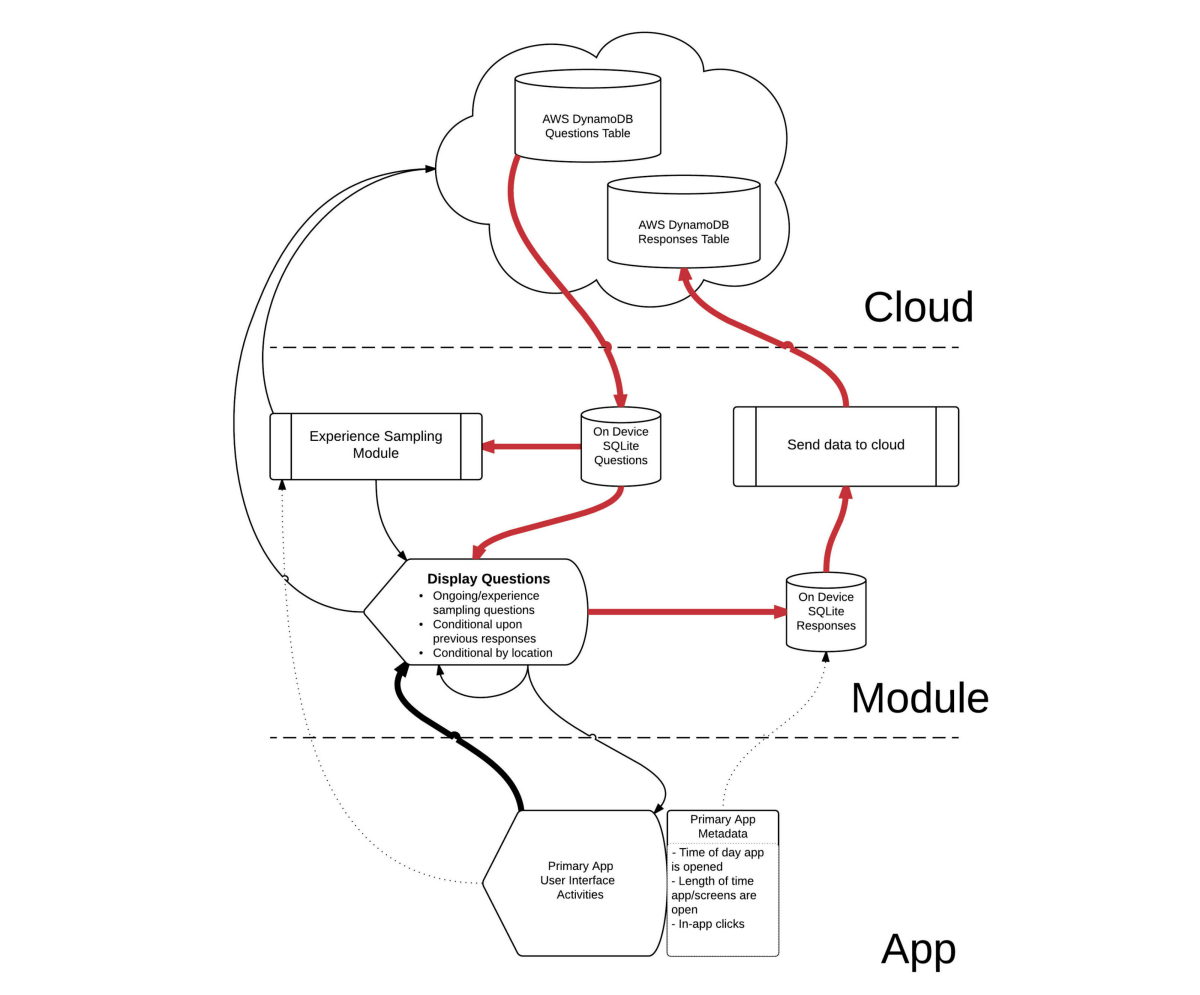
Overview of the Survalytics platform architecture. Black arrows are calls between subclasses of the module. Red arrows demonstrate data flow. Dotted lines serve to clarify the functionality and data storage existing in the original app, in the Survalytics module, and in the cloud database. The software module may be called by the primary app in several different ways. For surveys, the app should send a call to display survey questions. The app may make a different call to store arbitrary analytic data in the cloud. Finally, if desired, the app can call the experience sampling submodule. AWS: Amazon Web Services.

**Figure 2:**
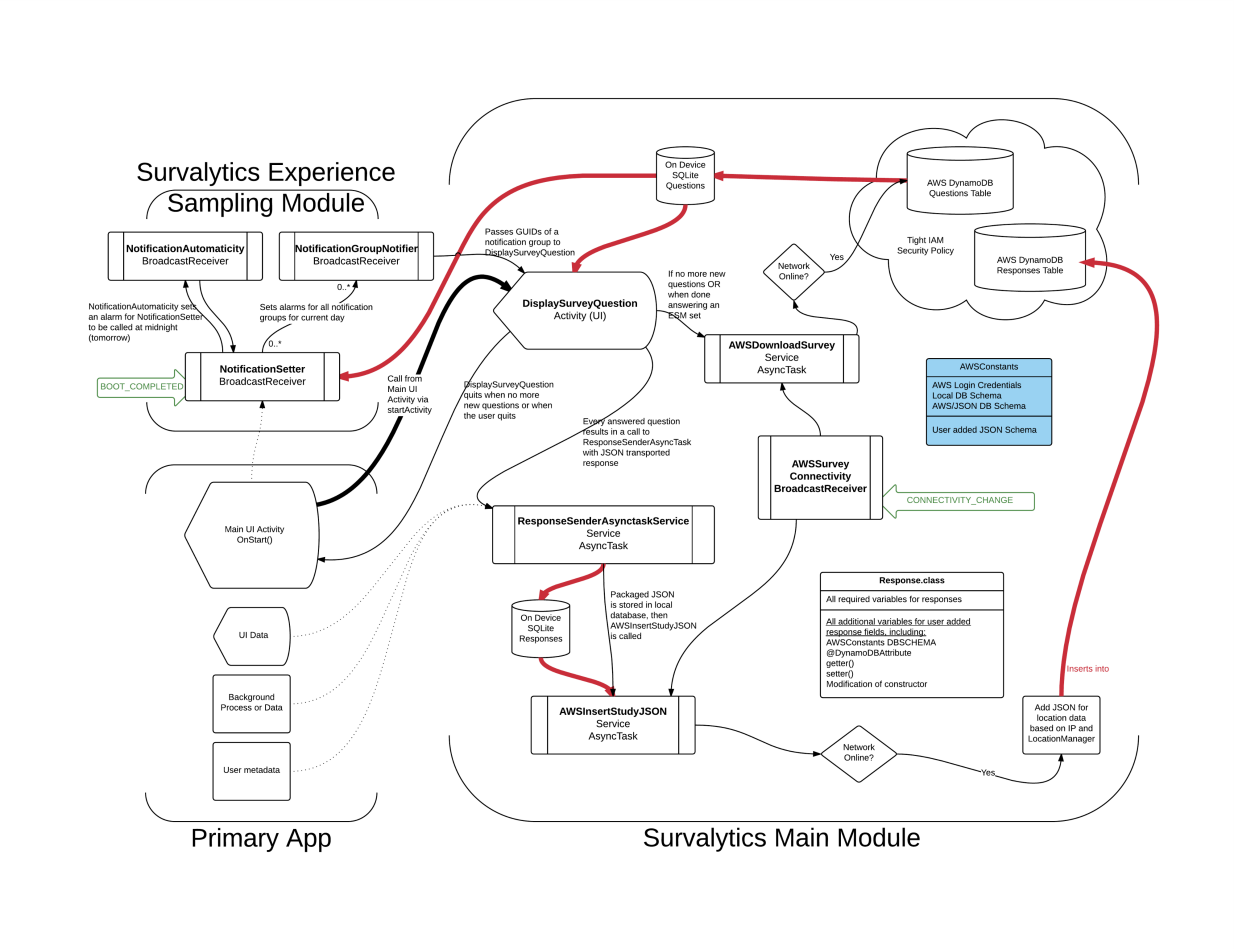
Detailed Survalytics platform architecture. The calls to be made from the primary app to the specific Java classes of the module are detailed here. In addition, the internal relationships between the classes of the module are also detailed. Black arrows are calls and red arrows demonstrate data flow. Light green arrows represent system level events that drive calls to the indicated classes. AWS: Amazon Web Services; DB: database; ESM: experience sampling module; IAM: Identity and Access Management; IP: Internet protocol address; JSON: JavaScript object notation; SQL: structured query language; UI: user interface. See Multimedia Appendix 5.

We have assembled the basic metadata about Survalytics in Table 1 and listed the required AWS software development kit (SDK) components in Textbox 1. The module is designed as a set of core (Table 2) and supplemental (Table 3) Java classes with minimal user modification required in order for the module to function. These modifications are straightforward and allow the high degree of flexibility for the various applications that this module will likely serve. Figure 1 provides an overview of the life cycle of the module, and Figure 2, Multimedia Appendix 5 provides the details of the interactions between the Java classes. Black arrows signify the calls between classes, and red arrows signify the flow of data into or out of databases. Light green arrows indicate OS-level broadcast events that are received by the module to activate background activity.

In order to get the software compiled and running, the only *required* modification is for users of the module to provide AWS credentials and table data in AWSConstants.java (as public static final variables). Multimedia Appendix 1 provides a walk-through of the process for establishing an AWS account, creating the 2 required DynamoDB tables (for Questions and for Responses), and creating an unauthenticated identity pool with strict security limitations. In particular, this identity pool is strictly limited to reading the Questions table and to writing single responses to the Responses table. Multimedia Appendix 2 provides a walk-through of the process for formatting and inserting questions into the Questions table, and Multimedia Appendix 3 provides a set of sample questions. The schema used is described in Multimedia Appendices 2 and, section I.

**Table 1:** Code metadata.

Descriptor	Description
Current code version	v1.0
Permanent link to code	http://dx.doi.org/10.15139/S3/12126
Legal Code License	Apache 2.0
Code versioning system used	None
Software code languages, tools, and services used	Java, Amazon Web Services (AWS) DynamoDB, Eclipse Mars IDE^a^
Compilation requirements, operating environments & dependencies	Eclipse Mars IDE Android SDK Target SDK >=9 (GINGERBREAD) AWS SDK for Android
Support email for questions	voreill@emory.edu

^a^IDE: integrated development environment.

**Required Amazon Web Services Android SDK JAR files (tested with version 2.2.5).**

aws-android-sdk-cognito-x.x.x.jar

aws-android-sdk-core-x.x.x.jar

aws-android-sdk-ddb-x.x.x.jar

aws-android-sdk-ddb-mapper-x.x.x.jar

**Table 2:** Core Survalytics Java classes.

Names	Notes
SA_AWSConstants.java	Required modification: AWS^a^credentials
SA_AWSDownloadSurvey.java	Pulls Questions into local DB^a^
SA_ResponseSenderAsynctaskService.java	Packages Responses and stores in local DB for later upload
SA_AWSInsertStudyJSON.java	Pushes Responses to AWS
SA_DisplaySurveyQuestion.java	Displays Questions
SA_QuestionDB.java	Wrapper class for all local DB calls
SA_Question.java	AWS DynamoDB Annotated Question object
SA_Response.java	AWS DynamoDB Annotated Response object
SA_AWSSurveyConnectivityBroadcastReceiver.java	Background Question download and Response upload with connectivity change

^a^AWS: Amazon Web Services; DB: database.

**Table 3:** Supplemental Survalytics Java classes.

Names	Notes
SA_AWSQuestionVerificationAndDisplay.java	Question Display and Validation Should not be included in production release of application
SA_NotificationAutomaticity.java SA_NotificationGroupNotifier.java SA_NotificationSetter.java	Survalytics Sampling: add-on classes for ecological momentary assessment (experiential sampling)

Only 1 of the core classes—DisplaySurveyQuestion—must be called by the end user to achieve survey functionality (dark arrow from the main user interface (UI) activity). All of the other classes provide background functionality for the module. DisplaySurveyQuestion is an Activity that displays questions present in the local database and also initiates a download of questions (red data flow arrow) from the cloud AWS table if no new questions are available. The activity recursively cycles through unanswered questions until no relevant questions remain. The end user has the option to delay answering questions until later (“Not now/answer later” button, easily removed from the UI if investigators do not wish participants to be able to delay answering).

In terms of analytics, as indicated in Figure 1 by dotted lines, users of the module may also optionally store additional types of data such as UI clicks, user inputted data, app metadata, or otherwise processed data using a direct call to ResponseSenderAsyncTaskService. The Responses class should be modified under these circumstances. In the study of the “Anesthesiologist” app mentioned in the Introduction section, we use this analytics storage component to track a variety of app uses, including total time using the app (per launch) and a variety of in-app clicks. This allows fine-grained analysis of the app usage linked directly to the survey responses collected, as described above, using DisplaySurveyQuestion.

Whether storing survey or analytics data, the data are stored on-device before attempting upload. If the device is offline, data are persisted in a private SQLite database on-device until the device comes online. The Android OS security model prevents access to these private SQLite databases by other apps and is only otherwise accessible by physically connecting a rooted, unlocked Android device to a computer. The change in connectivity is detected by the package (Figure 2, Multimedia Appendix 5) and upload of all stored data is initiated at that time.

Multimedia Appendix 4 contains the full JSON schema for the app and AWS tables and demonstrates the modifications to Responses required in Section IV. However, it should be noted that all data packaged into JSON for upload are transmitted to the final AWS Responses table in the “json_str” field. Therefore, even without modification to Responses, the data will remain preserved. This comes at the price of doubling the stored data; because storage is cheap and data are precious, this prevents situations where incorrect modification of Responses leads to loss of data. In this situation, users of the module will need to extract the JSON from the AWS Responses table to get at the data. Eventually, the AWS SDK for Android will likely include functionality to allow insertion of arbitrary JSON directly into DynamoDB tables, already a feature of the AWS SDK for Java [33]. This should obviate the need for Responses and for preventative double storage.

### Illustrative Example

Users may follow one of the many available guides for the setup of an Android integrated development environment (IDE) [34,35]. Survalytics was coded and tested using the Eclipse IDE [36]. Android Studio [37] is an IDE provided by Google for Android development, and the use of the module in that environment may require modifications to the module other than those described in the Multimedia Appendix 4.

After setting up the AWS DynamoDB tables as outlined in Multimedia Appendix 1 and inserting questions into the Questions table as outlined in Multimedia Appendix 2, the project should be loaded into the selected development environment and modifications made to AWSConstants.java to include the AWS security credentials gathered in Multimedia Appendix 1. The package also relies on the Android SDK for AWS [37,38]. Once downloaded, the 4 required Java Archive (JAR) files in Textbox 1 should be copied into the *libs* folder of the project. At this point, the project may be compiled and deployed onto a test or emulated device.

Important permissions and declarations are included in the Android manifest file and should be made note of when using Survalytics as an add-on module. These include permissions for Internet connectivity and use of the Global Positioning System and declarations for all the Survalytics classes. Any projects making use of the Survalytics must include these permissions and declarations in the Android manifest for proper functionality.

The package may be tested using the questions provided in Multimedia Appendix 3, modeled on the types of questions that might be asked in a study of blood glucose monitoring in diabetic patients. The Survalytics Question Builder for Google Sheets provides a simple template that will take the desired values for *questionguid_str*, *ordinalposition_int*, and so on and export JSON for easy insertion into the AWS Questions table. Users may validate the insertion of questions in the AWS Questions table using AWSQuestionVerificationAndDisplay, as discussed below.

The example app and questions provide insight into how the various functionalities of the module may be tied together to create a comprehensive study framework. Upon loading, an AlertDialog requesting consent for participation in the study is displayed. Acceptance or rejection of consent is uploaded to the AWS Responses table with entrytype “consentcode.” This is an example of direct upload of app UI data. Questions 10-40 are questions that are asked only once and collect basic demographic information about the study patients and their diabetes management. Question 100 is an ongoing experience sampling question asked daily at 11 am and 6 pm. Questions 101 and 110 illustrate the deletion of ongoing question 100 and insertion of the same ongoing question, except asked daily at 7 pm and then at 12 noon on Sunday only. Question 50 illustrates the use of *delaybydays* while question 60 illustrates the use of *conditional-upon-questionguid* and *conditional-upon-responseid*. The question will only be asked if question 40 (*questionguid* “DM1-FOLLOWUP”) was answered with *responseid* 1 (“Monthly”). Questions may also be *conditionalbycountry* ; this should be a comma-delimited list of International Organization for Standardization 3166 alpha-2 country codes listing the countries in which the question should be asked.

A basic survey visualization tool is included with Survalytics. This is the AWSQuestionVerificationAndDisplay class. As demonstrated in the example app, we recommend including this class as a menu option during survey development and app testing. This class presents the entire survey present in the AWS Questions table for inspection. While processing the questions, it performs a series of validation checks, including uniqueness of *ordinalposition*, validity of *conditional-upon-guid* and each *conditional-upon-responseid*, validity of each *notificationtime*, and validity of *deletequestion*. All elements of the question, including conditionals by time or country, are displayed. In the case where questions are *conditional-upon-guid*, each of the *conditional-upon-responseid* is matched to the *responseid_int* of the original question and those *response_str* are displayed, allowing at a glance verification of branch points. The menu option and the class itself should be removed from the production version of the app.

## Discussion

As indicated in the Introduction section, this project was motivated by the need for a tool with broad functionality allowing data capture across several domains. The flexible data model offered by NoSQL database solutions simplifies the approach to database management and data capture. By combining survey data with in-app usage analytics, we can capture detailed usage patterns of mobile Android apps and parse that behavior based on the collected demographics. As mentioned, the module will be deployed as part of a large research study of an international population of anesthesia providers. This study may shed important light on global practice patterns and areas for practice improvement in the global or in targeted communities. Most intriguingly, the module can serve not just to ask questions but also to deliver payloads of educational content to targeted end users based conditionally on their responses, location, and so on.

The stand-alone experience sampling functionality fills a substantial need in that it is the first cloud-integrated open-source experience sampling program available for deployment on a modern smartphone platform. The uses of such a program span a wide variety of fields, from sociology to economics to psychology to the biomedical sciences. As mentioned, existing solutions either are expensive and closed-source or lack the broad functionality presented here. The previous unreleased port of the Experience Sampling Program (ESP) to Android, presented in a poster session to the American Society of Anesthesiologists, generated several unsolicited requests from interested research groups. Survalytics should be a welcome addition to the experience sampling community.

One major advantage of Survalytics over existing packages is the use of AWS free-tier offerings. Amazon Web Services has a comprehensive security model (termed Identity and Access Management, or IAM), allowing extremely fine-grained permissions to be granted to various users. For example, users can have limited read and write capabilities down to the table level. Data security was a prime consideration in choosing AWS over other possible solutions; stand-alone servers may go without security updates in the absence of a mature IT support framework, and the other major NoSQL platform, MongoDB, offered very little in the way of granular security. Survalytics uses so-called “unauthorized entities” to access questions and write responses, but these “unauth identities” cannot write to the questions table or read the responses table if set up according to the instructions in Multimedia Appendix 1. All communication using the AWS SDK is, by default, secure sockets layer (SSL) encrypted. Access to data is limited by the account holder, and secondary users may be defined by that account holder using IAM with limited permissions. Certain AWS services are designed with Health Insurance Portability and Accountability Act (HIPAA) and personally identifiable information (PII) compliance in mind; DynamoDB is one of them. Interested parties may execute business associate agreements with AWS to use their services in a HIPAA compliant manner [39].

Using AWS carries some other significant advantages. There are mature SDKs available for a variety of platforms, including iOS, opening the possibility of developing Survalytics for other platforms (discussed below). Amazon Web Services offers a number of other free or low-cost services that may eventually be integrated into the Survalytics platform, including cloud computing and long-term archival services.

Although the Survalytics package as offered provides all the essential tools required to deploy the functionalities provided, several avenues of development remain. A data exploration tool for basic exploratory data analysis and visualization of the AWS Responses table would be valuable, in particular for users with a substantial analytics component or for those users collecting large datasets. Additionally, a more integrated approach to question building and upload may be helpful for those working with large surveys or multiple survey sets. The on-device language is currently detected by Survalytics, but there is no current mechanism to localize delivered questions on the basis of this language. This would be straightforward to implement but not a current area of need.

A major area of development is to port Survalytics to the iOS platform. We initially focused on developing the platform for Android OS as Android devices are profoundly cheaper than iOS devices, allowing researchers to deploy Survalytics at much lower equipment costs. While iOS devices have a higher market share in developed countries, Android continues to maintain parity with iOS globally. Our planned deployment of Survalytics for study of the “Anesthesiologist” app, currently only available for Android, led us to initially focus on the Android OS version of Survalytics. We are also actively exploring development of a cross-platform version of Survalytics using Xamarin.

Hopefully, contributions in some of these areas will emerge from the open-source user community, although development of tools for the projects we currently have in progress will only serve to further develop the Survalytics package for all users. Exploratory data analysis using the open-source statistical package R [40] is straightforward to implement using the jsonlite [41] library for import.

Efforts were made to optimize the end-user experience while maximizing code efficiency, but the user interface, which relies on the basic set of views and components provided by the Android OS, may be improved upon by contributors with experience and expertise in UI. There are almost certainly areas where the codebase could be optimized to reduce resource utilization or increase speed. On the other hand, the code was written explicitly adopting a philosophy in which simplicity of codebase took precedence over bandwidth or storage considerations. Since the module is targeted for use by nonexperts and experts alike, this approach improved the readability of the code. This accessibility should result in a larger number of researchers adopting the package as well as contributing to its further development.

## Appendix

Multimedia Appendix 1 Amazon Web Services walk-through.

Multimedia Appendix 2 Question formatting and insertion.

Multimedia Appendix 3 Sample questions table.

Multimedia Appendix 4 JSON and database schema.

Multimedia Appendix 5 Detailed Survalytics platform architecture.

## Abbreviations

AWS: Amazon Web Services
HIPAA: Health Insurance Portability and Accountability Act
IT: information technology
JAR: Java Archive
OS: operating system
UI: user interface

## References

[1] Gartner.

[2] Gartner Says Worldwide Traditional PC (2014). Gartner.

[3] Number of apps available in leading app stores 2015 | Statistic Internet.

[4] O’Reilly-Shah V (2016). Anesthesiologist: Anesthesia equipment, physiology,dose calculator for Android OS. JMIR mHealth uHealth Internet.

[5] Mobile Surveys Internet.

[6] Looking for an Android Survey App?.

[7] Apptentive.

[8] Quora.

[9] Shiffman S, Stone AA (1998). Introduction to the special section: Ecological momentary assessment in health psychology. Health Psychology.

[10] Shiffman S, Stone AA, Hufford MR (2008). Ecological momentary assessment. Annu Rev Clin Psychol.

[11] Eckhoff RP, Kizakevich PN, Bakalov V, Zhang Y, Bryant SP, Hobbs MA (2015). A Platform to Build Mobile Health Apps: The Personal Health Intervention Toolkit (PHIT). JMIR Mhealth Uhealth.

[12] Barrett LF, Barrett DJ (2001). An Introduction to Computerized Experience Sampling in Psychology. Social Science Computer Review.

[13] Quantified Self - Self Knowledge Through Numbers Internet.

[14] Luciano JS, Cumming GP, Wilkinson MD, Kahana E (2013). The emergent discipline of health web science. J Med Internet Res.

[15] Loveday A, Sherar LB, Sanders JP, Sanderson PW, Esliger DW (2015). Technologies That Assess the Location of Physical Activity and Sedentary Behavior: A Systematic Review. J Med Internet Res.

[16] ResearchGate Questions.

[17] Runyan J, Steenbergh T, Bainbridge C, Daugherty DA, Oke L, Fry BN (2013). A smartphone ecological momentary assessment/intervention "app" for collecting real-time data and promoting self-awareness. PLoS One.

[18] Conner T (2015). University of Otago.

[19] SurveySignal.

[20] CS diario.

[21] LifeData.

[22] mEMA by ilumivu.

[23] IFormBuilder.

[24] movisensXS.

[25] HarvestYourData.

[26] Paco.

[27] Emotion Sense.

[28] AWARE Framework.

[29] funf.

[30] CBITs TECH Web Site.

[31] Amazon Web Services, Inc.

[32] O’Reilly-Shah V, Mackey S (2011). ResearchGate.

[33] Stack Overflow.

[34] im292fresh Instructables.

[35] Android Developers.

[36] Eclipse Foundation.

[37] Android Developers.

[38] Amazon Web Services, Inc.

[39] Amazon Web Services, Inc.

[40] R Core Team (2015). R: A language and environment for statistical computing Internet.

[41] Ooms J arXiv stat CO.

