# Supplementary material for: Survalytics: An Open-Source Cloud-Integrated Experience Sampling, Survey, and Analytics and Metadata Collection Module for Android Operating System Apps
Source: JMIR Mhealth Uhealth. 2016 Jun 3;4(2):e46. doi: 10.2196/mhealth.5397 (PMC4912681; doi:10.2196/mhealth.5397)
Supplement: Multimedia Appendix 1 [file mhealth_v4i2e46_app1.pdf]

Appendix A: Amazon Web Services Setup Walkthrough

1. Go to http://aws.amazon.com and click to sign up.

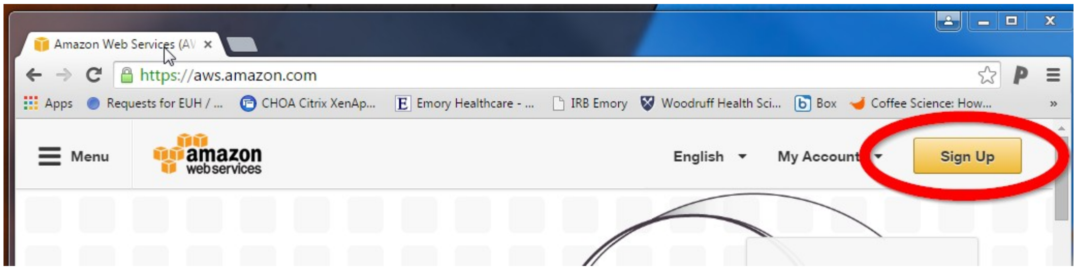

2. Enter your email address and indicate that you are a new user.

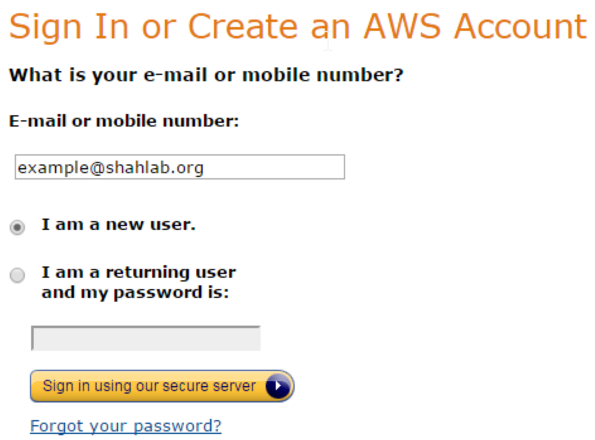

3. Enter your name, email address, and strong password. Warning: You are creating a live Amazon Web Services account that may be used by hackers to instantiate expensive and highly processor intensive activities such as BitCoin mining. Protect your credentials.

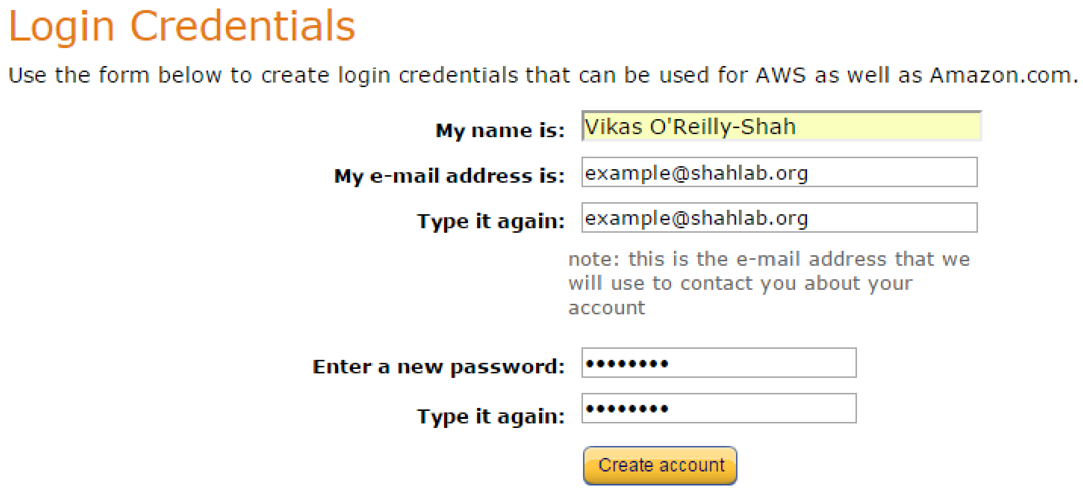

~~4. Enter your contact information.~~

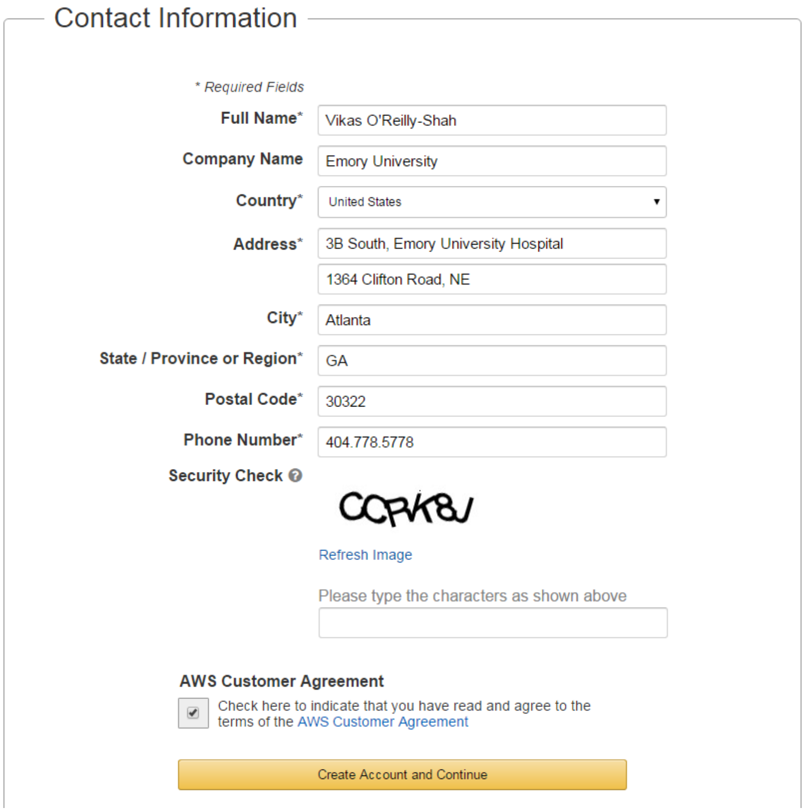

Contact Information

* Required Fields

Full Name*: Vikas O'Reilly-Shah

Company Name: Emory University

Country*: United States

Address*: 3B South, Emory University Hospital
1364 Clifton Road, NE

City*: Atlanta

State / Province or Region*: GA

Postal Code*: 30322

Phone Number*: 404.778.5778

Security Check

Refresh Image

Please type the characters as shown above

AWS Customer Agreement

Check here to indicate that you have read and agree to the terms of the AWS Customer Agreement

Create Account and Continue

~~5. Enter payment information. If you follow the instructions laid out in this tutorial, you will mostly likely remain in the free tier of service indefinitely. As explained below, free tier service includes a large amount of space and throughput for most Survalytics use cases.~~

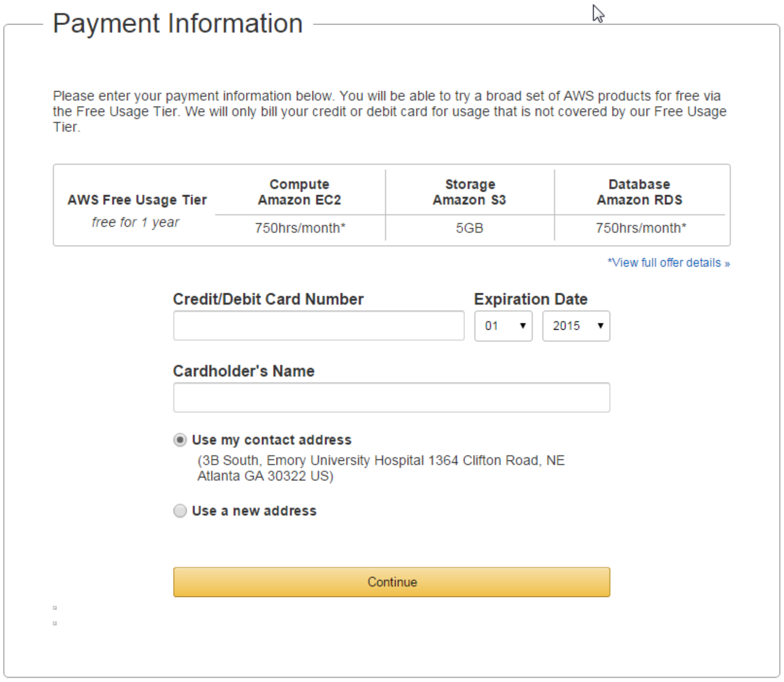

Payment Information

Please enter your payment information below. You will be able to try a broad set of AWS products for free via the Free Usage Tier. We will only bill your credit or debit card for usage that is not covered by our Free Usage Tier.

AWS Free Usage Tier	Compute Amazon EC2	Storage Amazon S3	Database Amazon RDS
free for 1 year	750hrs/month*	5GB	750hrs/month*

*View full offer details »

Credit/Debit Card Number

Expiration Date: 01 | 2015

Cardholder's Name

Use my contact address
(3B South, Emory University Hospital 1364 Clifton Road, NE Atlanta GA 30322 US)

Use a new address

Continue

~~6. Provide a live telephone number to perform identity verification.~~

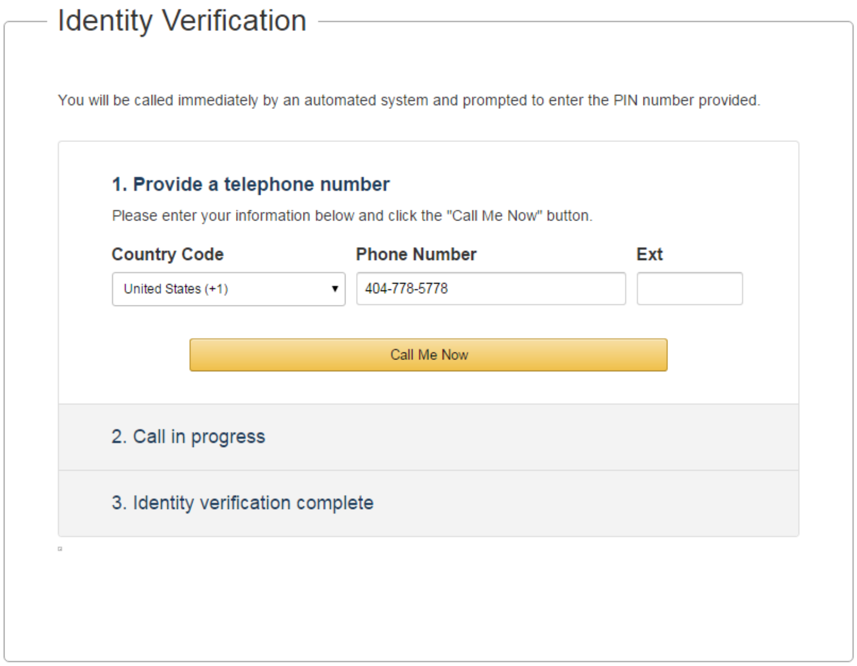

Identity Verification

You will be called immediately by an automated system and prompted to enter the PIN number provided.

1. Provide a telephone number

Please enter your information below and click the "Call Me Now" button.

Country Code	Phone Number	Ext
United States (+1)	404-778-5778	

Call Me Now

2. Call in progress

3. Identity verification complete

~~7. Sign up for the Basic support plan, which is free.~~

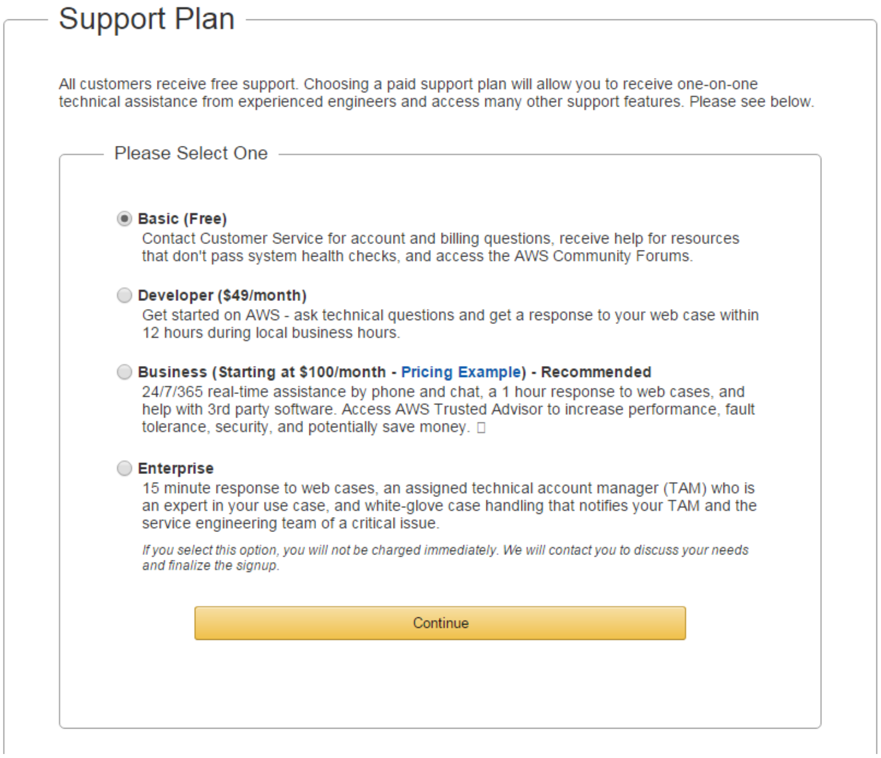

Support Plan

All customers receive free support. Choosing a paid support plan will allow you to receive one-on-one technical assistance from experienced engineers and access many other support features. Please see below.

Please Select One

Basic (Free)
Contact Customer Service for account and billing questions, receive help for resources that don't pass system health checks, and access the AWS Community Forums.

Developer ($49/month)
Get started on AWS - ask technical questions and get a response to your web case within 12 hours during local business hours.

Business (Starting at $100/month - Pricing Example) - Recommended
24/7/365 real-time assistance by phone and chat, a 1 hour response to web cases, and help with 3rd party software. Access AWS Trusted Advisor to increase performance, fault tolerance, security, and potentially save money.

Enterprise
15 minute response to web cases, an assigned technical account manager (TAM) who is an expert in your use case, and white-glove case handling that notifies your TAM and the service engineering team of a critical issue.

If you select this option, you will not be charged immediately. We will contact you to discuss your needs and finalize the signup.

Continue

~~8. Once sign-up is complete, you can sign in to the console using your credentials.~~

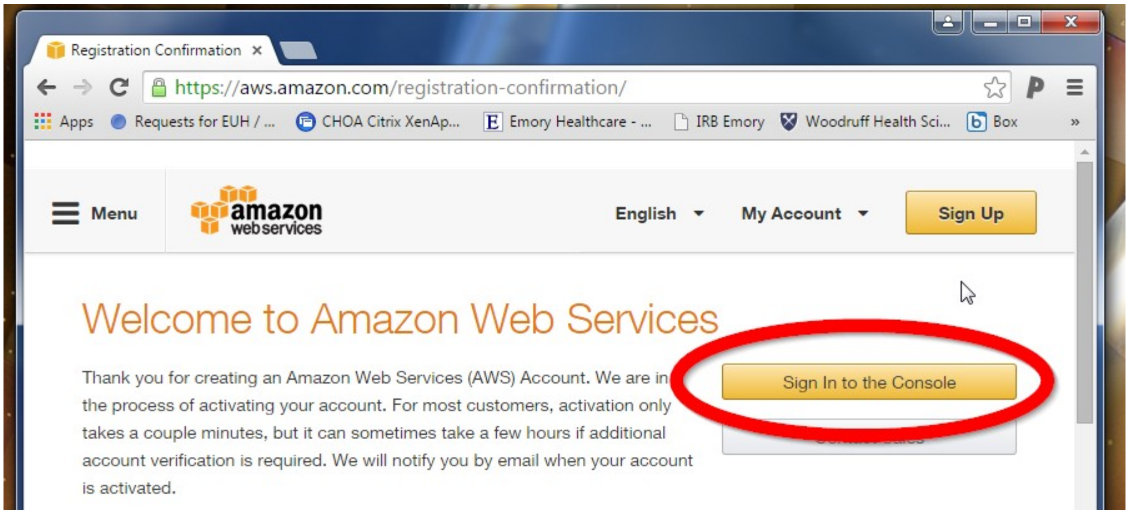

~~**9. Go to the DynamoDB service.**~~

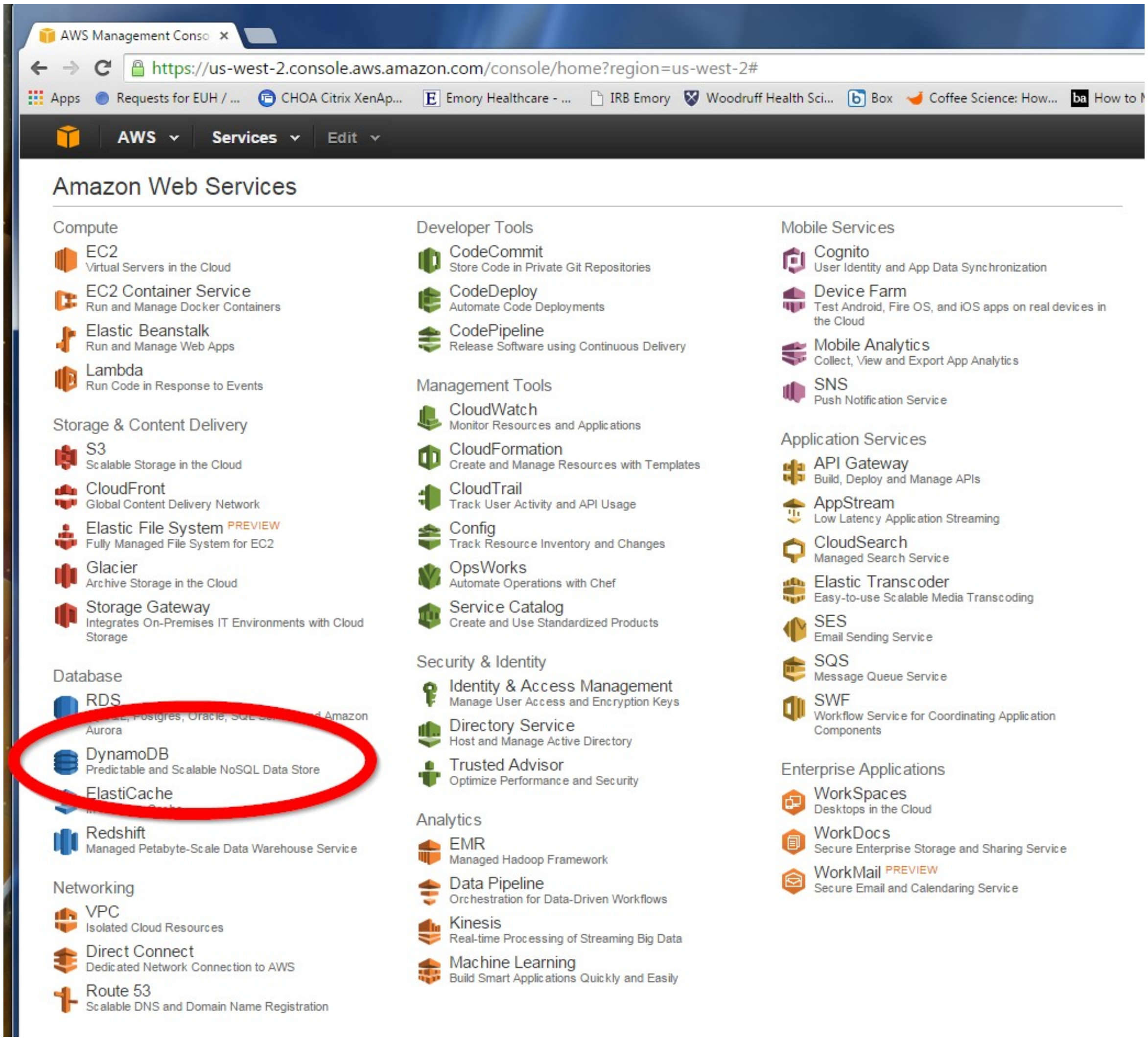

~~**10. Click on Create Table.**~~

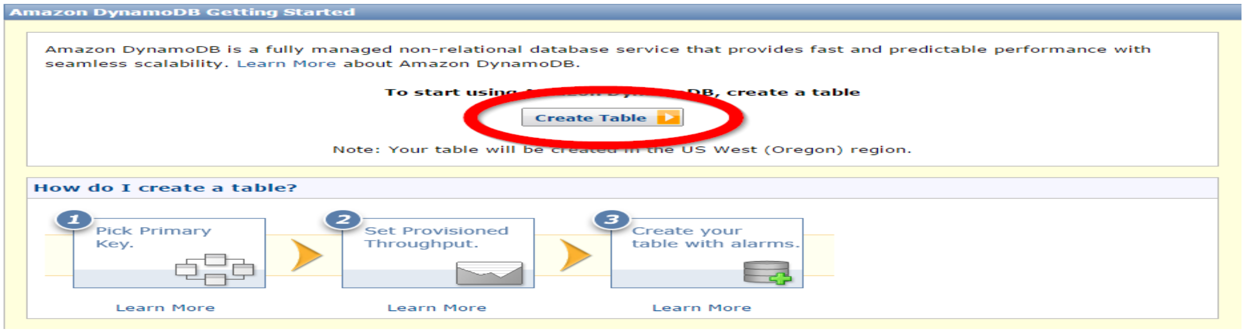

~~11. Create your table with the following options:~~

~~Name: survalytics_questions~~

~~Hash key: questionguid_str (String)~~

~~(HASH only, do not include a Range key)~~

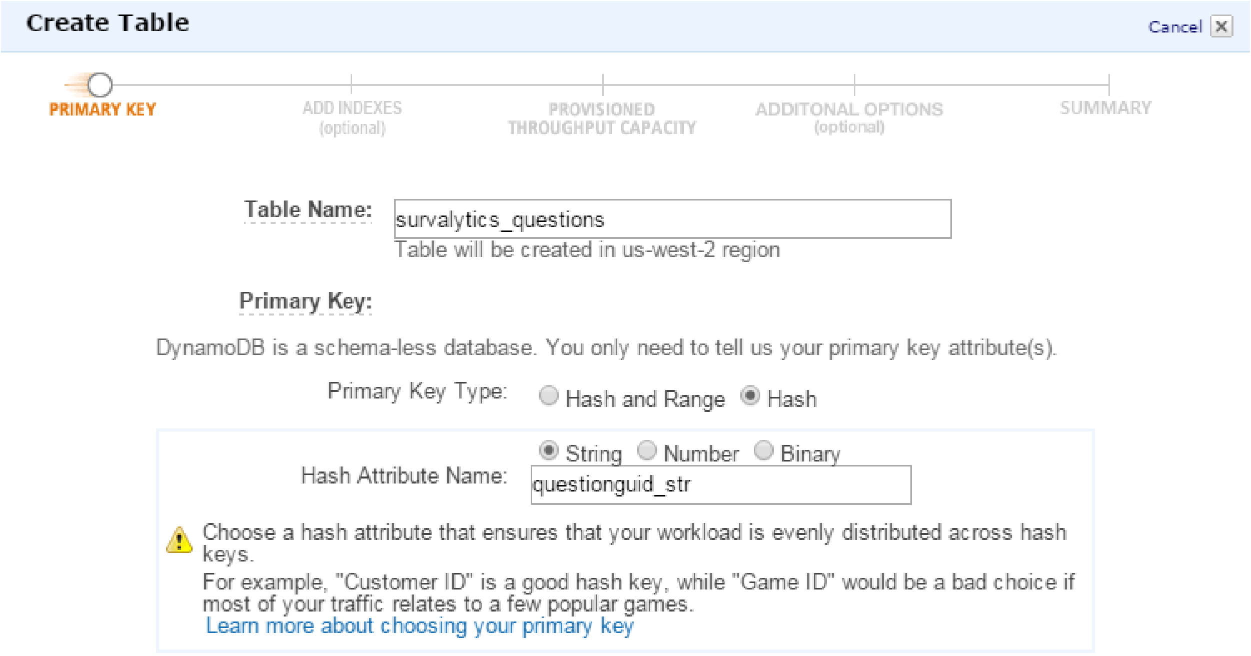

~~12. Click continue on the next screen. This table will not need secondary indices..~~

~~13. Provision throughput of 5 Read Units and 5 Write Units. Warning: You may not exceed a total of 25 Read Units and 25 Write Units across ALL your tables. If you do, you will be charged even if you do not put a single piece of data into a single one of your tables. Provisioning throughput is like signing up for a monthly plan from a cell provider. If you sign up, you pay for it, even if you don't use it.~~

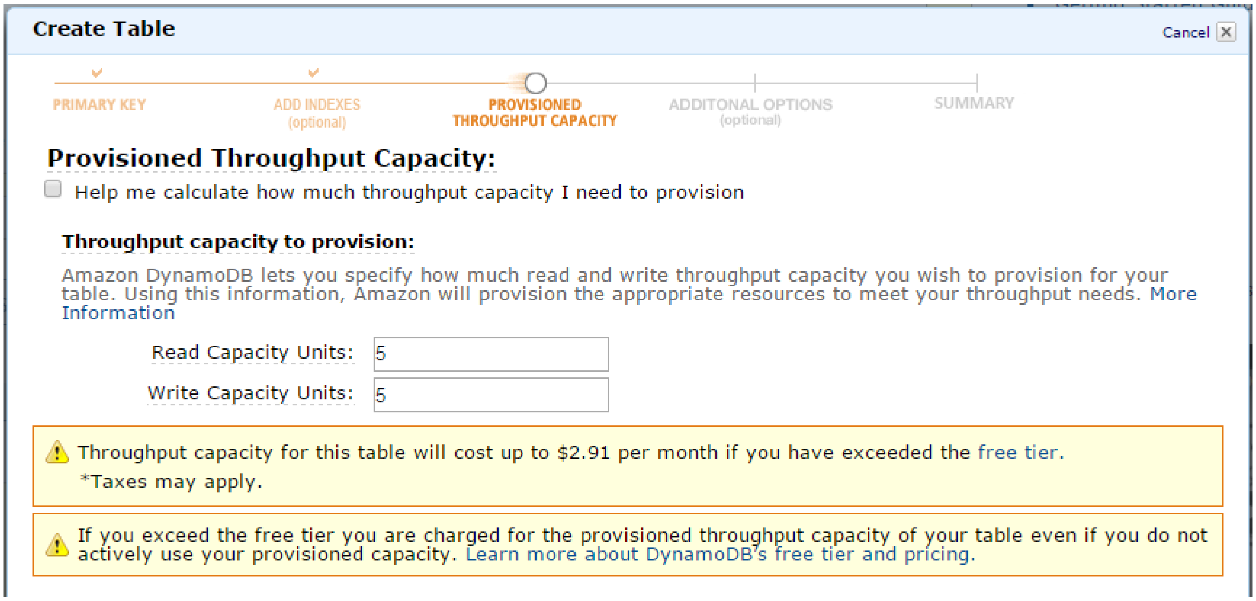

~~14. You may sign up for alarms if throughput thresholds are exceeded. Once in production, this may be valuable for wildly successful platforms and applications, as the provisioned throughput can be modified upward or downward at will.~~

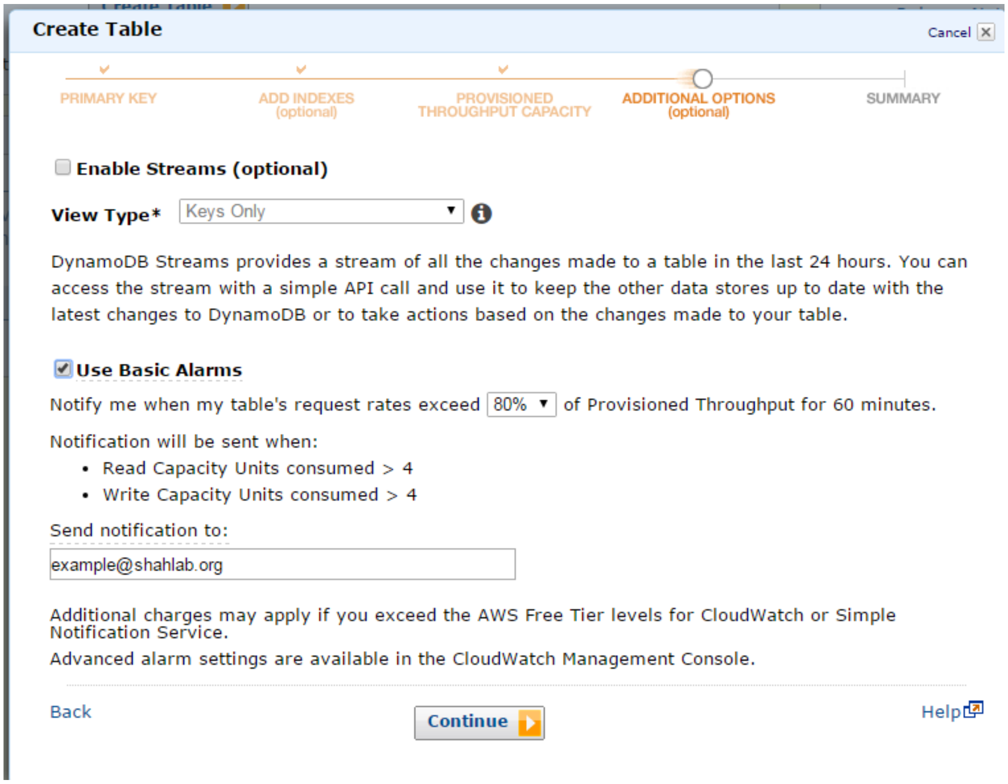

~~15. Click Create Table and, following the above process, create a table as below:~~

~~**NAME: SURVALYTICS_RESPONSES**~~

~~**HASH KEY: ENTRYTYPE_STR (STRING)**~~

~~**RANGE KEY: LOCALTIME_MS_INT (NUMBER - IF THIS IS SET INCORRECTLY THE APP WILL FAIL)**~~

~~**READ: 5**~~

~~**WRITE: 5**~~

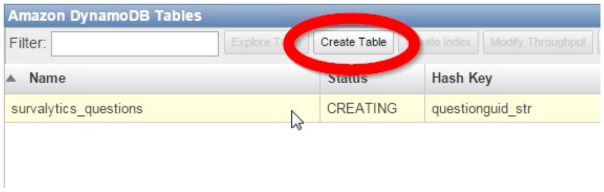

~~**16. COPY YOUR TABLE ARNS TO A TEXT FILE, AS YOU WILL NEED THEM LATER:**~~

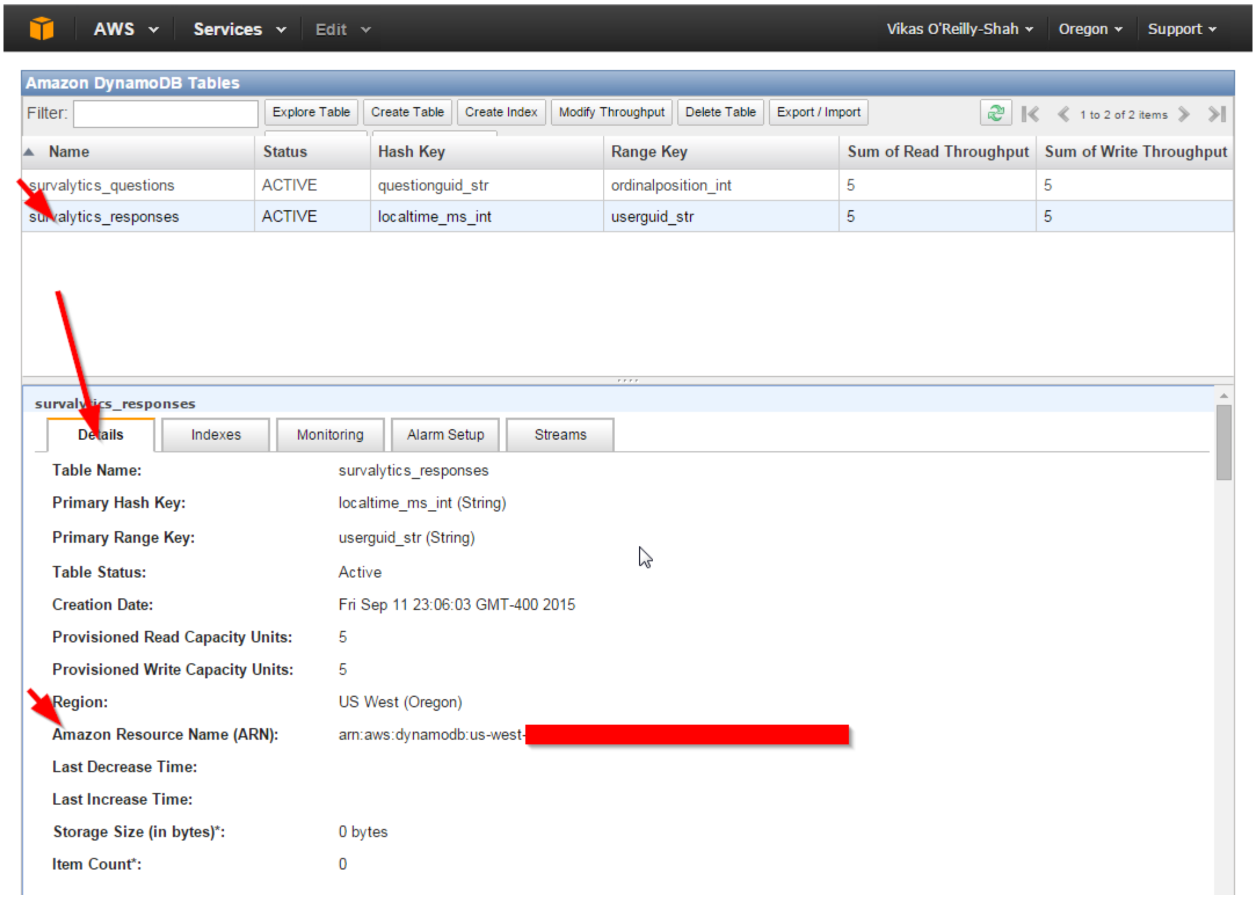

~~17. Now we will use AWS Identity and Access Management to create a very tight security policy which will be used by our application to access the questions and store the responses. Go to the Cognito service and click Get started. You may be prompted to change regions. IF SO, make a note of the region you are coming from. You DynamoDB tables only exist in that region. Then switch to one of the regions where Cognito is available (at the time of writing, US East and EU Ireland.) You will need to switch back to your originating region to see the tables you previously created.~~

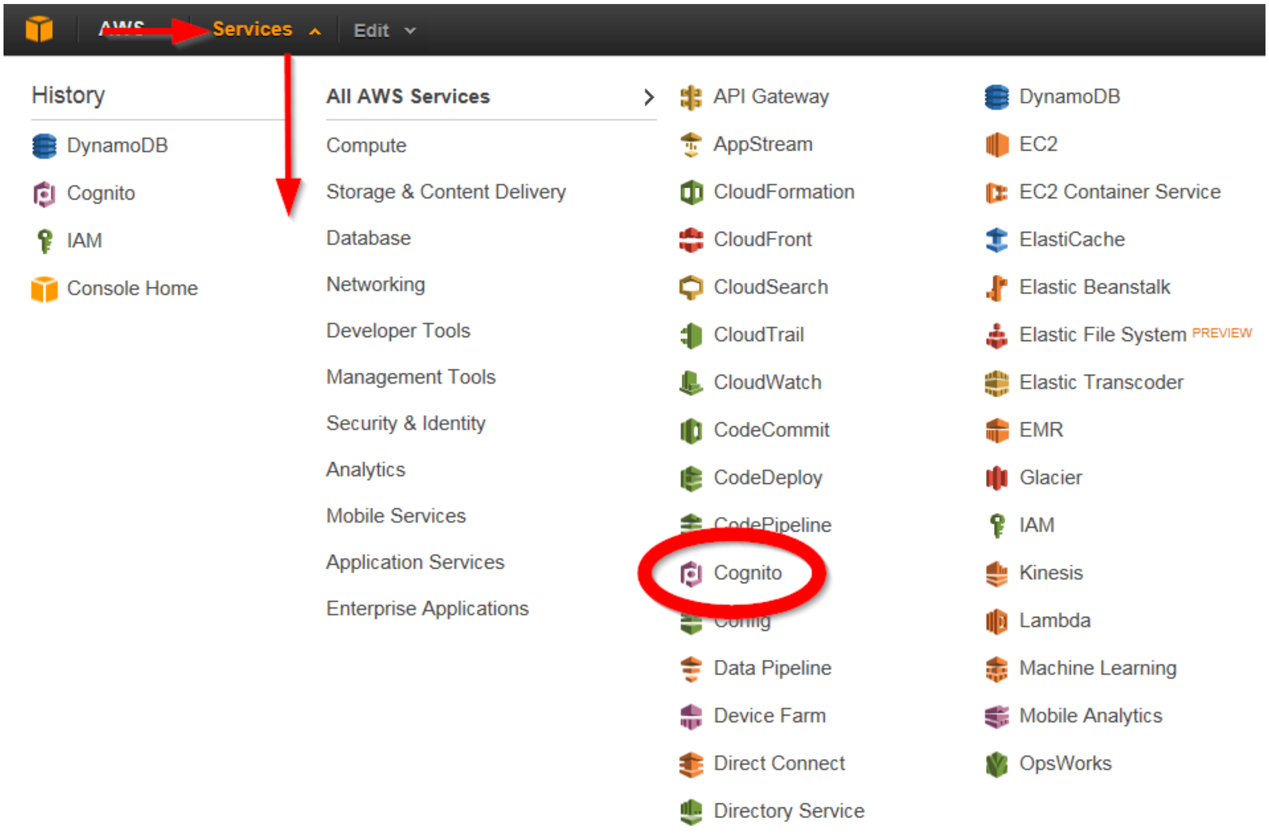

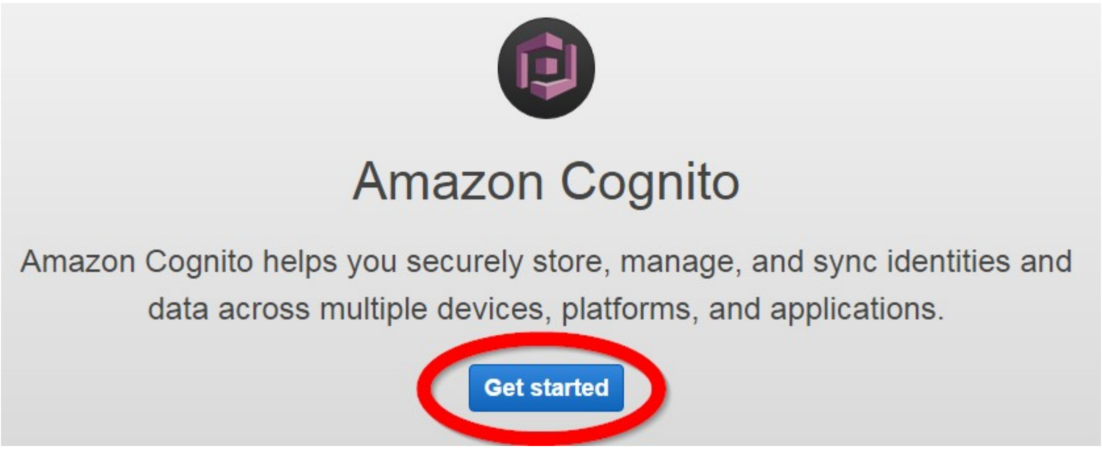

~~18. Name your identity pool. Ensure that unauthenticated identities are allowed. Click Create Pool and, on the next screen, Allow.~~

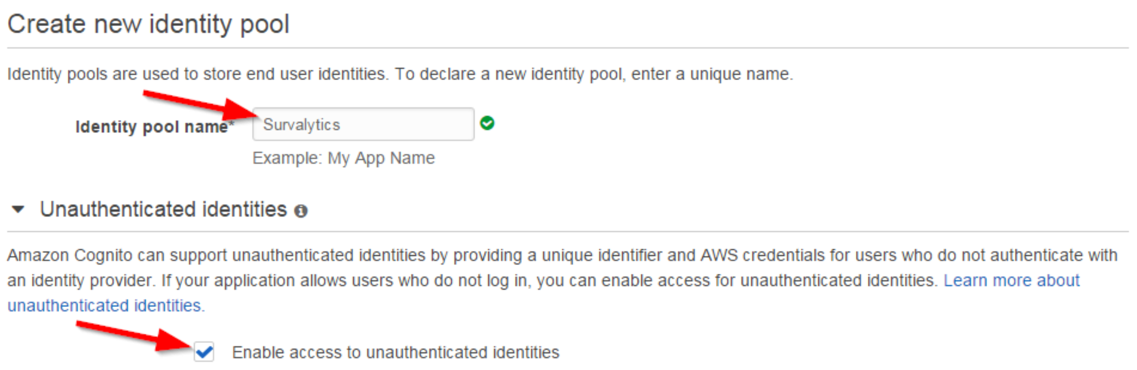

~~19. Click Edit identity pool.~~

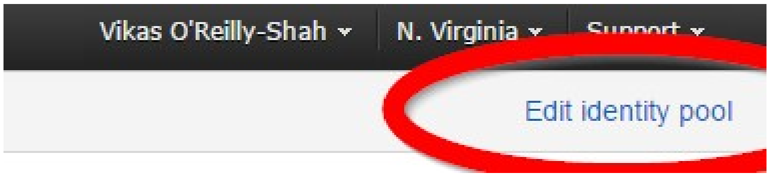

~~20. Copy your identity pool ID into a text file. You'll need it later.~~

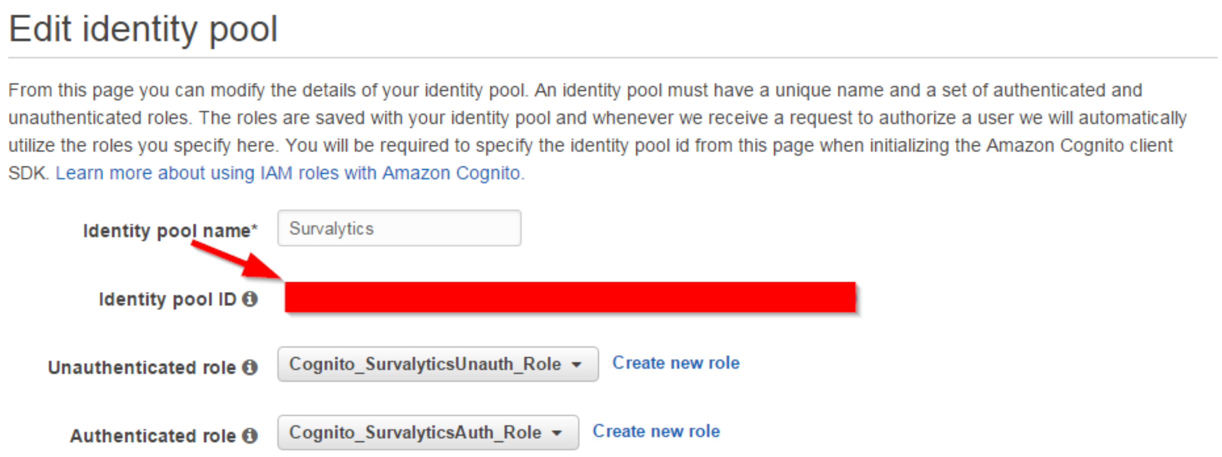

~~21. Go to the security credentials section.~~

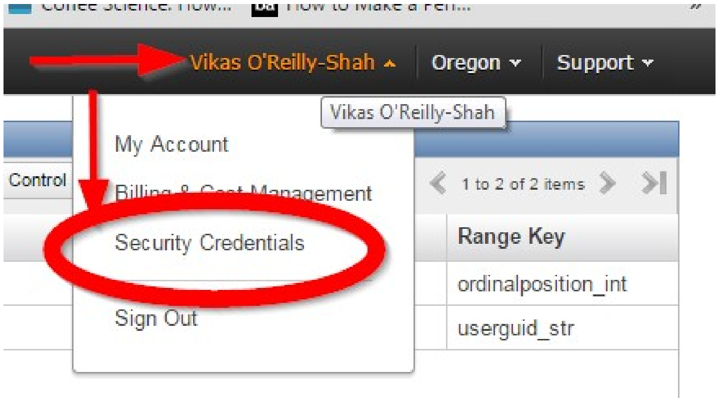

~~**22. Click on Policies. Click Create Policy.**~~

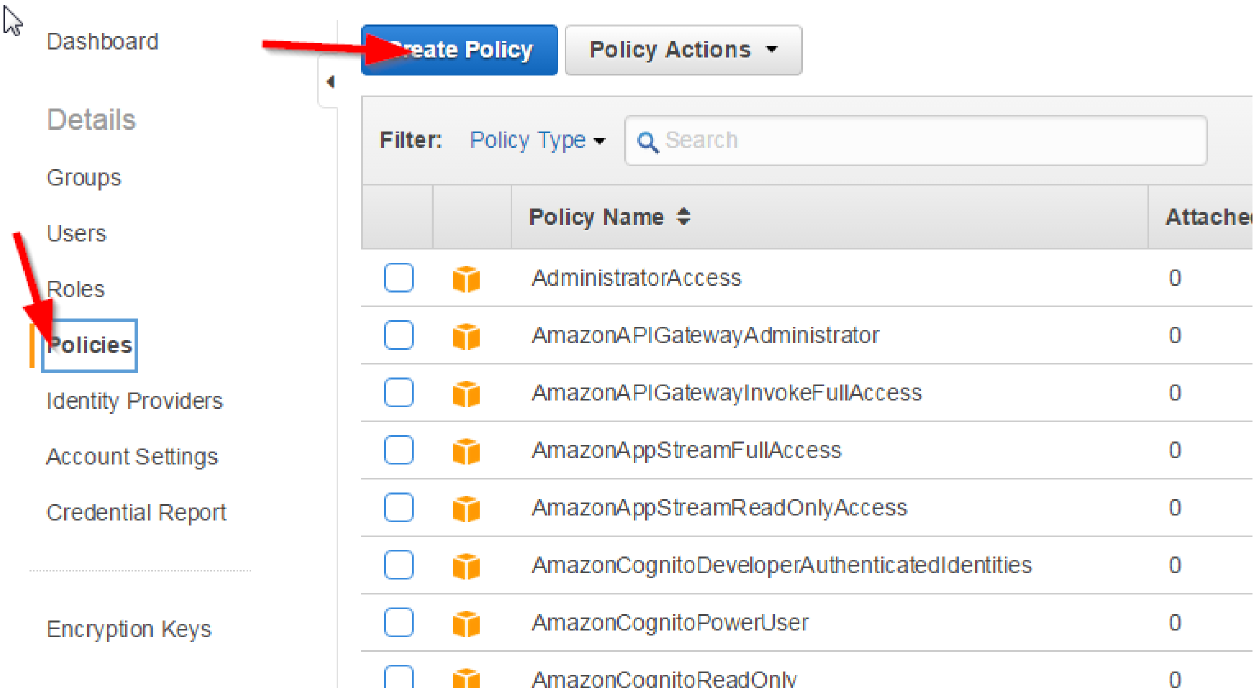

~~**23. Select the Policy Generator.**~~

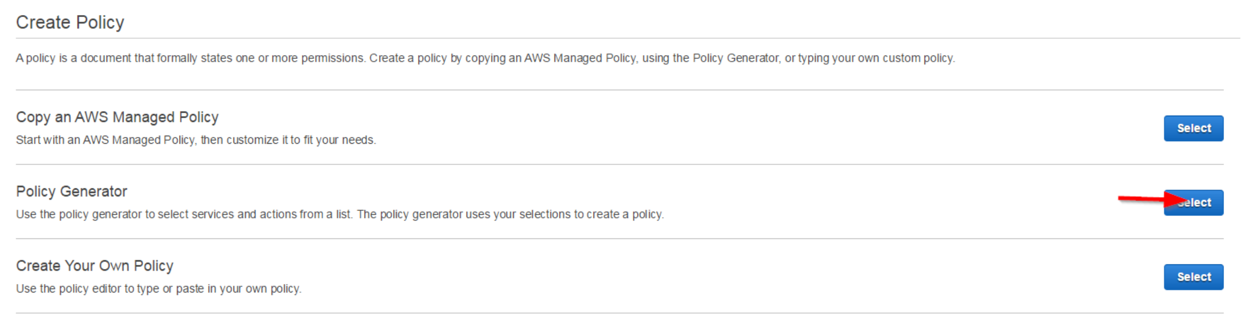

~~**24. Do the following:**~~
~~**Effect: Allow**~~
~~**AWS Service: DynamoDB**~~
~~**Actions: PutItem and UpdateItem ONLY**~~
~~**ARN: ~~~ Your Responses Table ARN ~~~**~~

~~Click Add Statement~~

~~Effect: Allow~~
~~AWS Service: DynamoDB~~
~~Actions: GetItem, BatchGetItem and Scan ONLY~~
~~ARN: ~~~ Your Questions Table ARN ~~~~~
~~Click Add Statement~~

~~Click Next Step~~

~~Policy Name: Survalytics~~
~~Description: Survalytics~~

~~Click Create Policy~~

~~25. Scroll down to Survalytics policy and click on it.~~

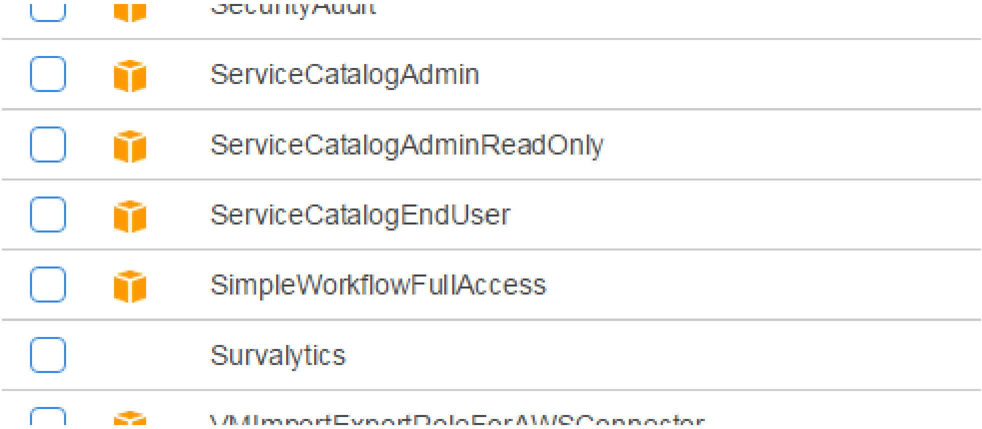

~~26. Click Attach under Attached Entities~~

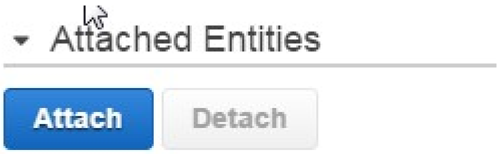

~~27. Check CognitoSurvalytics_Unauth and click Attach Policy below.~~

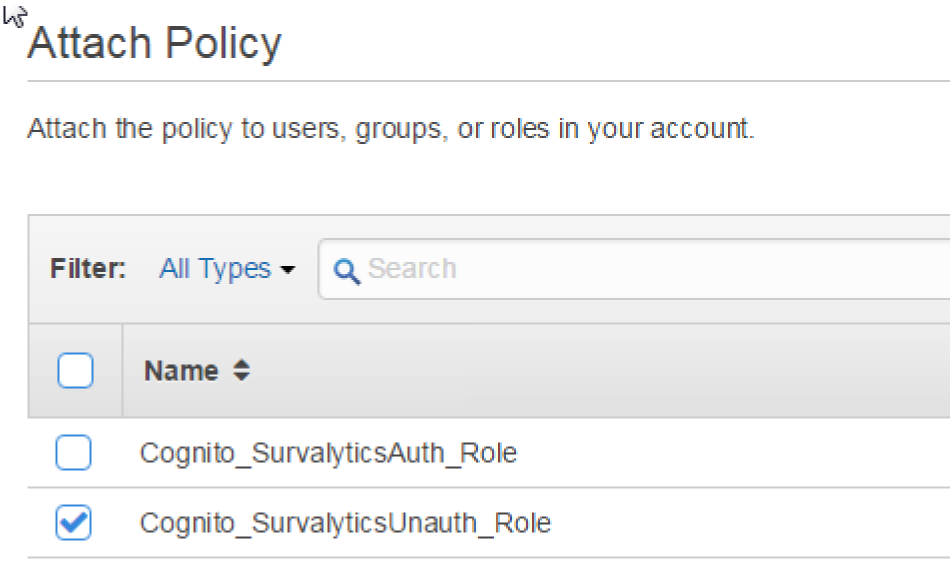
Attach Policy
Attach the policy to users, groups, or roles in your account.
Filter: All Types
Search
Name
Cognito_SurvalyticsAuth_Role
Cognito_SurvalyticsUnauth_Role

28. Copy the unauth ARN to a text file:

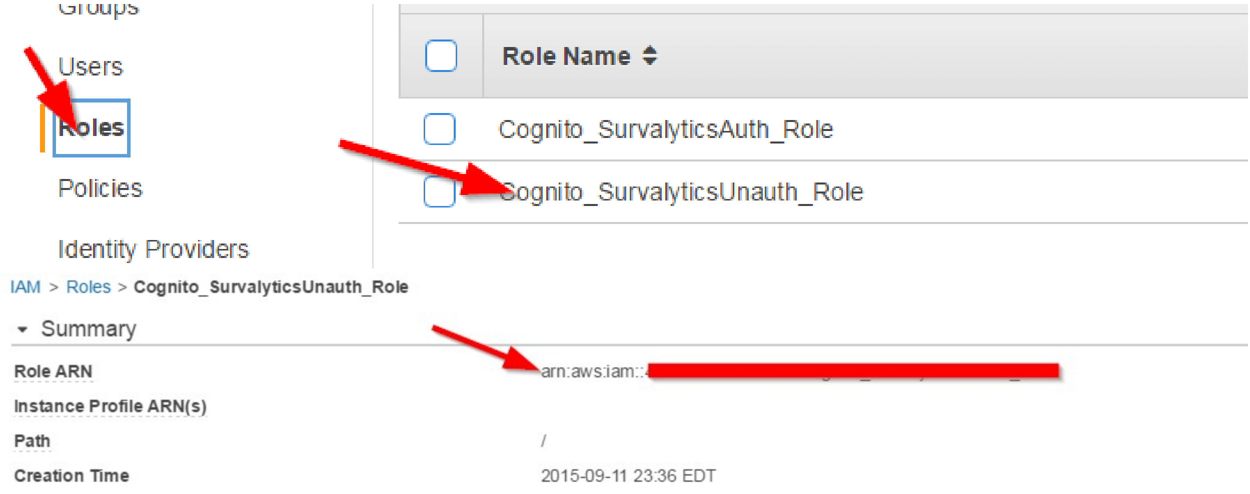

29. Copy your account ID to a text file.

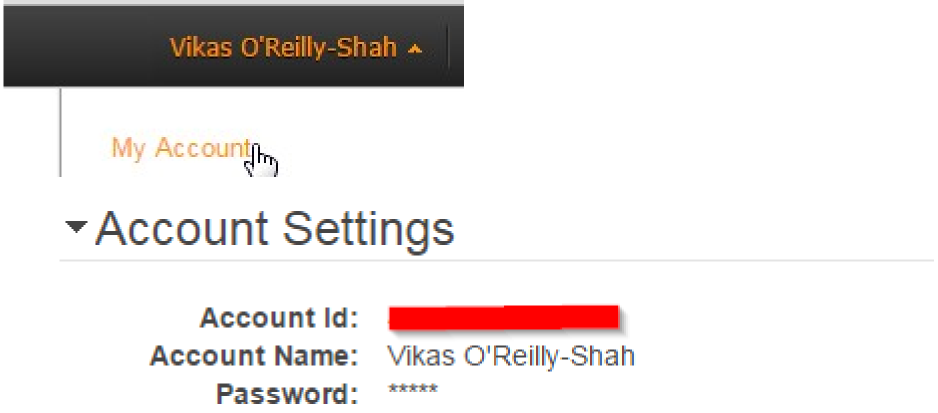

At this point you will have the following pieces of information, necessary to put into SA_AWSConstants.java:

```
public static final String ACCOUNT_ID = "~~~~~";
//Identity pool ID only below, do not put full ARN
public static final String IDENTITY_POOL_ID = "~~~~~";
public static final String UNAUTH_ROLE_ARN = "~~~~~";
//Table names only below, do not put full ARN
public static final String RESPONSETABLENAME = "~~~~~";
public static final String QUESTIONTABLENAME = "~~~~~";
```

Congratulations! You've set up a cloud database with a tight security policy that will work with the example application. Put the constants needed into the Java file above. Please see Appendix B for putting your first set of questions into the Questions table.
