# Supplementary material for: Survalytics: An Open-Source Cloud-Integrated Experience Sampling, Survey, and Analytics and Metadata Collection Module for Android Operating System Apps
Source: JMIR Mhealth Uhealth. 2016 Jun 3;4(2):e46. doi: 10.2196/mhealth.5397 (PMC4912681; doi:10.2196/mhealth.5397)
Supplement: Multimedia Appendix 2 [file mhealth_v4i2e46_app2.pdf]

Appendix B: Question Formatting and Insertion

Questions are formatted as a series of key-value pairs in a JSON formatted container. Several excellent overviews of the format are available online[1–3].

A Google Sheets based script is available to generate the required JSON for you allowing very easy insertion of questions into the AWS Questions table. All users of this module will need a Google account for deploying Android devices. Creating a Google account is very straightforward. Once created, go to your Google Drive at http://drive.google.com. Click New and then Google Sheets. Once in your new spreadsheet, click Tools then Script Editor. Delete any pre-existing code and copy/paste the contents of SurvalyticsQuestionToJSON.gs (in the assets folder of the Android source code package) into the Code.gs. Ctrl+S to save then close the window. Once you return to your spreadsheet, F5 to refresh the browser window. There will be a new option in the Toolbar: "Survalytics". The first option "Setup Question Template" will create a new sheet and set up the template for generating the JSON. The second option, "Export Question JSON", will generate the JSON you will copy/paste into AWS. JSON for export for multiple questions at the same time may be generated; to see how this works, copy ALL the example questions to template. The "~~~END~~~" tag must be present in column 1 for the JSON Generator to function properly. This tag is placed anywhere below your final question, ideally where your next surveyguid_str would go.

The process for inserting questions in the AWS Questions is straightforward. Log in to your AWS Console and go to DynamoDB. Ensure you are in the correct region. Double clicking on your Questions table will allow you to see existing entries and perform a number of tasks. Click Create Item. Switch from Tree to Text entry. Delete the existing JSON and copy the JSON from "Export Question JSON" into the text box. Click Save and you have inserted the question successfully. Tools for question validation and mapping will be forthcoming as this tool continues to improve and evolve.

Please see Appendix C for the entire database schema. The Question schema is described in detail below; this JSON Object has several required and optional key-values pairs. Table 1 provides a series of example questions. These questions are also found in the assets folder of the Survalytics package and may be imported into Google Sheets and directly copied line by line into the JSON generator.

surveyname_str and surveyguid_str: REQUIRED

Both are JSON String key-value pairs containing arbitrary text. No requirement for uniqueness. May be used to divide questions into groups subgroups for future querying.

ordinalposition_int: REQUIRED

JSON Integer key-value pair. Must be a unique positive integer within the question set. Determines the order in which questions are presented to the end user.

questionguid_str: REQUIRED

JSON String key-value pair containing arbitrary text. Must be a unique String comprised of numbers, letters, and dashes only. Provides a unique value for querying Responses.

questionprompt_str: REQUIRED

JSON String key-value pair containing arbitrary text. No requirement for uniqueness. Prompt displayed to the end user.

questiontype_str: REQUIRED

~~JSON String key-value pair containing one of the following four constants: "buttons", "text", "checkboxes", or "slider". Specifies the nature of the collected data.~~

~~responses_arr: REQUIRED~~

~~JSON Array containing an ordered set of JSON Objects. Each JSON Object must have two key-value pairs: responseid_int and response_str. The questiontype_str above determines the handling of the response Objects.~~

~~"buttons" and "checkboxes":~~

~~Each object represents a single response displayed to the end user as an option. responseid_int is a JSON Integer that is appended to questionguid_str in the stored response. response_str is the JSON String that is the response prompt displayed to the end user.~~

~~"text"~~

~~Only the first response Object is used. response_str provides the "hint" shown in the textbox. reponseid_int is unused.~~

~~"slider"~~

~~Only the first two response Objects are used. response_str must be an integer in each of these Objects. They define the lower and upper bound of the slider. reponseid_int is unused.~~

~~conditional_upon_questionguid_str and conditional_upon_responseid_arr: OPTIONAL~~

~~Joint optional JSON String key-value pair and JSON Array of JSON Integer key-value pairs. Both must be present. If present, Survalytics will query the local on-device database for the presence of the question with questionguid_str that equals conditional_upon_questionguid_str. The handling then depends on the original questiontype_str.~~

~~"buttons"~~

~~Tests whether each conditional_upon_responseid_int in the array conditional_upon_responseid_arr is equal to final_responseid_int. If so, the question will be displayed in the order determined by its ordinalposition_int above.~~

~~"checkboxes"~~

~~Tests whether each conditional_upon_responseid_int in the array conditional_upon_responseid_arr is equal any of responses checked. If so, the question will be displayed in the order determined by its ordinalposition_int above.~~

~~"slider"~~

~~There must be two and only two conditional_upon_responseid_int in the array conditional_upon_responseid_arr. These ints define the inclusive lower bound (first item in the JSONArray) and upper bound (second item in the JSON Array) for the response given to the original question with the slider. If the response is within those bounds, the question will be displayed in the order determined by its ordinalposition_int above.~~

~~conditionalbycountry_str: OPTIONAL~~

Optional JSON String key-value pair. If present, Survalytics will examine the country the end user is in based on TelephonyManager, LocationManager, and ipapi.com. If any of these three ISO 3166 alpha-2 country codes are present in the comma delimited list of these codes contained in conditionalbycountry_str, the question will eventually be displayed to the user based on the question's ordinalposition_int and other conditionals present in the question. Otherwise the question is not even inserted into the local on-device database.

conditional_upon_datemsid_int: OPTIONAL

Optional JSON Integer key-value pair. If present, Survalytics will not display the question until after conditional_upon_datemsid_int is greater than the Unix epoch time in milliseconds. Most Survalytics deployments will prefer to use delaybydays_int instead.

delaybydays_int: OPTIONAL

Optional JSON Double key-value pair. Survalytics looks for the presence of this flag when the question is first downloaded from the AWS Questions table. If present, Survalytics calculates (# of milliseconds in a day) * (delaybydays_int) + System.currentTimeMillis() and adds conditional_upon_datemsid_int to the question json_str. Thus, it delays the display of that question by the set number of days. This is useful for preventing survey fatigue by spacing questions out, or for asking for feedback after a set period of time from the initialization of the Survalytics package. NOTE: Originally coded to accept integer values only, now OK to use fractional as well as integral numbers of days.

ongoingquestion_arr: OPTIONAL

Optional JSON Array containing an ordered set of JSON Objects. Each JSON Object has one key-value pair: notificationtime_str, which is a specifically formatted JSON String. The String is composed of the three letter abbreviation for notification day of week (Mon, Tue, Wed, Thu, Fri, Sat, Sun OR DLY for daily) followed by the four digits specification of the notification time in 24 hour format (0000-2359). Examples include Mon1200, Dly0900. Other optional flags are ignored if ongoingquestion_arr is present. (No support for conditional ongoing questions at this time.)

deletequestion_str: OPTIONAL

Optional JSON String key-value pair. If present, Survalytics will query the local on-device database for the presence of the question with questionguid_str = deletequestion_str and, if present, delete that question. Allows deletion of ongoing questions that have already been deployed to end users.

References

1. JSON [Internet]. [cited 25 Sep 2015]. Available: http://www.json.org/

2. JSON Tutorial [Internet]. [cited 25 Sep 2015]. Available: http://www.w3schools.com/json/

3. tutorialspoint.com. JSON Syntax. In: www.tutorialspoint.com [Internet]. [cited 25 Sep 2015]. Available: http://www.tutorialspoint.com/json/json_syntax.htm
