# Supplementary material for: Survalytics: An Open-Source Cloud-Integrated Experience Sampling, Survey, and Analytics and Metadata Collection Module for Android Operating System Apps
Source: JMIR Mhealth Uhealth. 2016 Jun 3;4(2):e46. doi: 10.2196/mhealth.5397 (PMC4912681; doi:10.2196/mhealth.5397)
Supplement: Multimedia Appendix 4 [file mhealth_v4i2e46_app4.pdf]

Appendix C: JSON and Database Schema

I. Survey/demographics central database tables

The overall architecture is designed to simplify the codebase by using JSON primarily as a transport vehicle and limiting the number of database fields to those that need to be known by the database in question. For example, the AWS source database for downloading questions only needs to know questionguid (for a hash key) and the json_str containing the meat of the question. Telling it ordinal position simplifies other areas of the Android code and so that was included. Otherwise, the content remains unparsed until downloaded by the Android app.

On device, the database is again limited to guid, ordinal position, and jsonstr. The additional fields are flags for internal tracking use. Parsed JSON supplies fields for the generation of the question on-device and for the uploaded response.

http://www.jsoneditoronline.org/
https://www.guidgenerator.com/online-guid-generator.aspx

AWS Question Table:

```
        questionguid_str          : STRING, PRIMARY HASH KEY
        ordinalposition_int       : INT, RANGE KEY
        json_str                  : STRING
```

json_str JSON Schema: Question

```
{
        surveyname_str: STRING
        surveyguid_str            : STRING
        ordinalposition_int       : INT
        questionguid_str          : STRING
        questionprompt_str        : STRING
        questiontype_str          : STRING[1]
        responses_arr             : ARRAY
                [
                        {
                        responseid_int  :INTEGER
                        response_str            :STRING
                        },....
                ]
```

[1] public static final String TYPE_BUTTONS = "buttons";
public static final String TYPE_TEXT = "text";
public static final String TYPE_CHECKBOXES = "checkboxes";
public static final String TYPE_SLIDER = "slider";

OPTIONALLY

CONDITIONAL_UPON_QUESTIONGUID_STR : STRING // QUESTIONGUID TO CHECK*
CONDITIONAL_UPON_RESPONSEID_ARR : ARRAY// RESPONSEIDS TO CHECK*
[
{
CONDITIONAL_UPON_RESPONSEID_INT:INTEGER
}, …
]

CONDITIONALBYCOUNTRY_STR : STRING // USE ISO 3166 ALPHA-2 CODES

CONDITIONAL_UPON_DATEMSID_INT : INTEGER
// DATE (IN UTC UNIX EPOCH MS) AFTER WHICH TO ADMINISTER THIS QUESTION

DELAYBYDAYS_INT : INTEGER
//WAIT THIS MANY DAYS AFTER THE QUESTION IS FIRST DOWNLOADED TO ASK THIS QUESTION

ONGOINGQUESTION_ARR : ARRAY //ARRAY OF DAY OF WEEK+TIME AS FOLLOWS
[
{
NOTIFICATIONTIME_STR : STRING
}, …
]
//NOTIFICATIONTIME FORMATTED AS FOLLOWS: EEEHHMM
// EEE = THREE LETTER DAY OF WEEK (MON, TUE, WED, THU, FRI, SAT, SUN, DLY)
// DLY = DAILY
// HH = HOURS 00-23
// MM = MINUTES 00-59
// EXAMPLES: TUE0900, THU1400, DLY1200

DELETEQUESTION_STR : STRING //QUESTIONGUID OF ONGOING QUESTION TO
// DELETE FROM LOCAL SQLITE DB
}

LOCAL DB ON ANDROID

TABLE QUESTIONS
QUESTIONGUID_STR
JSON_STR
ORDINALPOSITION_INT
FINAL_RESPONSEID_INT
FINAL_RESPONSE_STR
ANSWERED_BOOL

TABLE RESPONSES
_ID
JSON
UPLOADED

II. Responses: Generic response schema

The generic schema serves as the basic information passed with all types of uploaded data. The additional overhead is minimal and the presence of this information in each of uploaded packet simplifies future analysis against unnecessary complexity in terms of secondary lookups. Most NoSQL solutions do not allows JOINs.

```
{
        userGuid_str                    : String      //
        localTime_ms_int                : Integer     //Primary Range Index[2]
        localTime_hrsMilitary_int       : Integer
        localTime_dayOfWeek_str         : String
        localTimeZone_str               : String
        country_tm_str                          : String      //from Telephony Manager
        lo_lang_str                     : String      //Locale lang
        region_ipapi_str                : String      //www.ip-api.com/json
        regionName_ipapi_str            : String
        country_ipapi_str               : String      //from ipapi
        region_gc_str                   : String      //from Geocoding
        country_gc_str                          : String      //from Geocoding
        latitude_gc_float               : Float       //easily excluded using boolean
        longitude_gc_float              : Float       //easily excluded using boolean
        latLonAccurary_gc_float         : Float       //easily excluded using boolean
        entryType_str                   : String      //Primary Hash Index
        ...
}
```

2 GUID unique to the installation, which allows a unique user's behavior over time to be analyzed while making it practically impossible the identify that user

III. Responses: Specific added fields to generic document schema

Questions

```
        …
        entryType_str           : "survey",
        surveyGuid_str          : STRING
        questionGuid_str        : STRING
        questionPrompt_str      : STRING
        response_str            : STRING
        responseId_str : STRING //questionGuid_str & "-" Integer.toString(responseId_int)
        responses_arr           : ARRAY [if type is multiple response eg checkbox)
                [
                        {
                        responseId_str :STRING
                                //questionGuid_str & "-" Integer.toString(responseId_int)
                        response_str            :STRING
                        },
                        {
                        responseId_str :STRING
                                //questionGuid_str & "-" Integer.toString(responseId_int)
                        response_str            :STRING
                        },
                        ....
                ]
```

Consent/consent change

```
        …
        entryType_str                   : "consentCode_int/consentChange_int"
        consentCode_int                 : INTEGER
        consentChange_int               : INTEGER
                                                1 - do not consent
                                                2 - consent
                                                3 - exit study
                                                4 - re-enter study
```

On start

```
        …
        entryType_str                   : "onStart"
        age_yrs_fra                     : FRACTION
        weight_kg_fra                   : FRACTION
```

Total time using the app

```
        …
        entryType_str                   : "totalTimeOfUse",
        timeInApp_ms_int                : INTEGER,
```

IV. Modifying for new responses

In order to add response fields to the JSON document in the AWS Responses table, follow these steps:

1. Add the new field to the database schema in AWSConstants:

```
public static final String DBSCHEMA_MYNEWENTRYTYPE = "myentrytype";
public static final String DBSCHEMA_MYNEWFIELD = "my_new_field_str";
```

2. Make the call to ResponseSenderAsyncTaskService in your code:

```
JSONObject jc = new JSONObject();
String mydata = "mydata";
try     {
        jc.put(SA_AWSConstants.DBSCHEMA_ENTRYTYPE,
        SA_AWSConstants.DBSCHEMA_MYNEWENTRYTYPE);
        jc.put(SA_AWSConstants.DBSCHEMA_MYNEWFIELD,
        mydata);
        } catch (JSONException e) {}
Intent c = new Intent(getBaseContext(), SA_ResponseSenderAsynctaskService.class);
c.putExtra(SA_AWSConstants.INTENT_EXTRA_JSONSTRING, jc.toString());
startService(c);
```

3. Add the variable for the data to Response.java. Create getter and setter including DynamoDB tag.
NOTE: If the data is numeric or boolean, use the nullable "Boolean", "Double" or "Integer" as opposed to the primitive "boolean", "double" or "integer".

```
private String mydata;
@DynamoDBAttribute
        (attributeName = SA_AWSConstants.DBSCHEMA_MYNEWFIELD)
        public String getmydata() {
                return mydata;
        }

        public void setmydata(String tmydata) {
                this.mydata = tmydata;
        }
```

4. Add the following conditional statement to the constructor Response(JSONObject j):

```
        if(j.has(SA_AWSConstants.DBSCHEMA_MYNEWFIELD)){
        userguid_str = j.getString(SA_AWSConstants.DBSCHEMA_MYNEWFIELD);
        }
```
