# Supplementary figures and images for: Survalytics: An Open-Source Cloud-Integrated Experience Sampling, Survey, and Analytics and Metadata Collection Module for Android Operating System Apps
Source: JMIR Mhealth Uhealth. 2016 Jun 3;4(2):e46. doi: 10.2196/mhealth.5397 (PMC4912681; doi:10.2196/mhealth.5397)

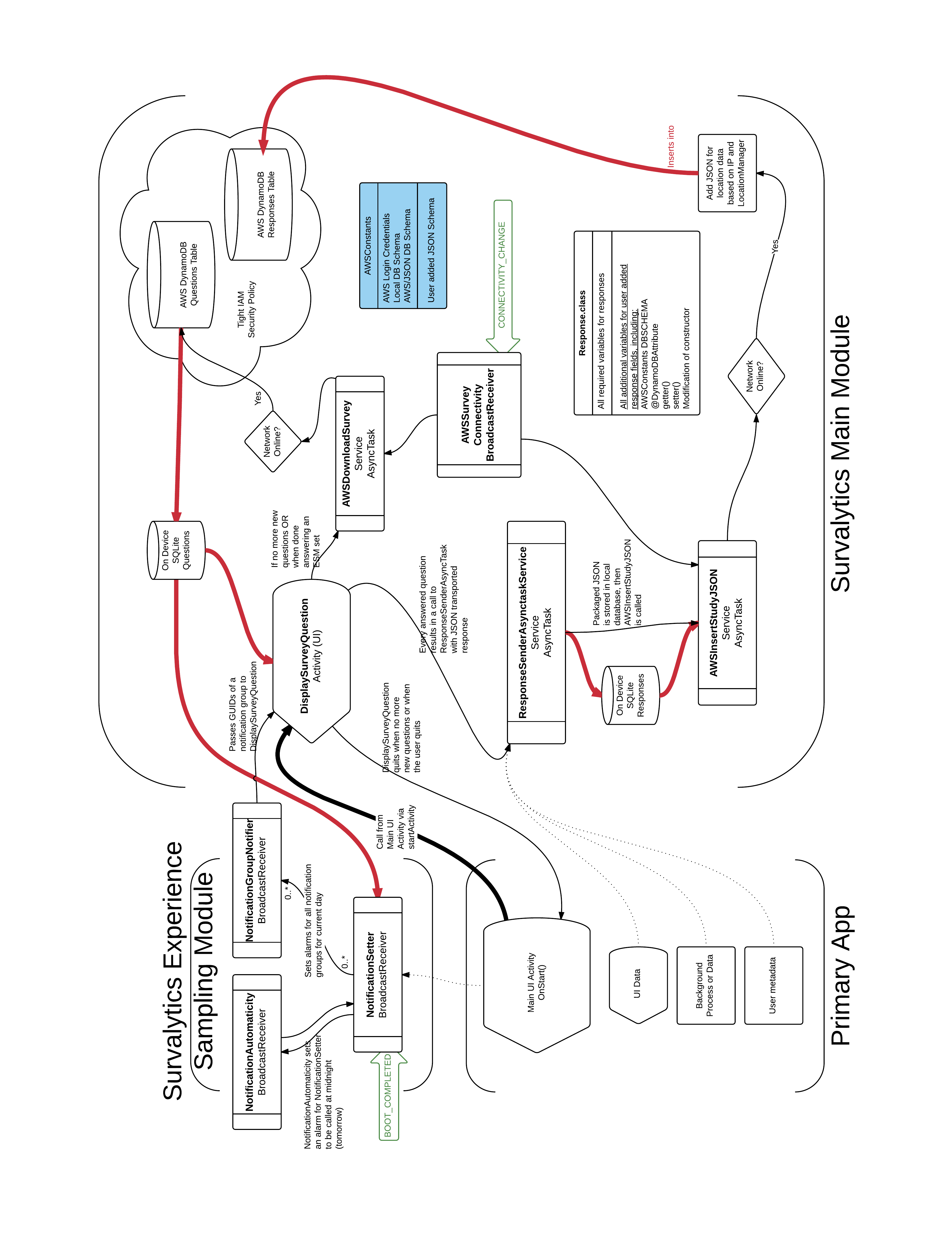

Supplement: Multimedia Appendix 5 [file mhealth_v4i2e46_app5.png]
